# ALKBH1 promotes HIF-1*α*-mediated glycolysis by inhibiting *N-*glycosylation of LAMP2A

**DOI:** 10.1007/s00018-024-05152-z

**Published:** 2024-03-12

**Authors:** Yanyan Liu, Mengmeng Li, Miao Lin, Xinjie Liu, Haolin Guo, Junyang Tan, Liubing Hu, Jianshuang Li, Qinghua Zhou

**Affiliations:** 1 Key Laboratory of Regenerative Medicine of Ministry of Education, The First Affiliated Hospital, Jinan University, Guangzhou, 510632 Guangdong China; 2 The College of Life Science and Technology, Jinan University, Guangzhou, 510632 Guangdong China; 3 The Biomedical Translational Research Institute, Health Science Center (School of Medicine), Jinan University, Guangzhou, 510632 Guangdong China

**Keywords:** ALKBH1, HIF-1*α*, Chaperone-mediated autophagy, *N-*glycosylation, Lysosome

## Abstract

ALKBH1 is a typical demethylase of nucleic acids, which is correlated with multiple types of biological processes and human diseases. Recent studies are focused on the demethylation of ALKBH1, but little is known about its non*-*demethylase function. Here, we demonstrate that ALKBH1 regulates the glycolysis process through HIF-1*α* signaling in a demethylase-independent manner. We observed that depletion of ALKBH1 inhibits glycolysis flux and extracellular acidification, which is attributable to reduced HIF-1*α* protein levels, and it can be rescued by reintroducing HIF-1*α*. Mechanistically, ALKBH1 knockdown enhances chaperone-mediated autophagy (CMA)-mediated HIF-1*α* degradation by facilitating the interaction between HIF-1*α* and LAMP2A. Furthermore, we identify that ALKBH1 competitively binds to the OST48, resulting in compromised structural integrity of oligosaccharyltransferase (OST) complex and subsequent defective *N-*glycosylation of LAMPs, particularly LAMP2A. Abnormal glycosylation of LAMP2A disrupts lysosomal homeostasis and hinders the efficient degradation of HIF-1*α* through CMA. Moreover, NGI-1, a small-molecule inhibitor that selectively targets the OST complex, could inhibit the glycosylation of LAMPs caused by ALKBH1 silencing, leading to impaired CMA activity and disruption of lysosomal homeostasis. In conclusion, we have revealed a non*-*demethylation role of ALKBH1 in regulating *N-*glycosylation of LAMPs by interacting with OST subunits and CMA-mediated degradation of HIF-1*α*.

**Supplementary Information:**

The online version contains supplementary material available at 10.1007/s00018-024-05152-z.

## Introduction

AlkB homolog 1 (ALKBH1), a 2-oxoglutarate and Fe^2+^-dependent hydroxylase has been proven to be implicated in nucleotide and histone demethylation [1]. ALKBH1 is capable of mediating the demethylation of various substrates, including DNA, mRNA, tRNA, and methylated lysine of histone H2A [2–5]. As a vital eraser of epigenetics, ALKBH1 is associated with numerous biological processes and human diseases, including embryonic development [6, 7], osteogenic and adipogenic differentiation [8, 9], chronic kidney disease and cancer [10–12]. Previous research has demonstrated that ALKBH1 can respond to the glucose availability [13]. However, the precise role and underlying molecular mechanisms pertaining to ALKBH1 in glycolysis remain to be elucidated.

Hypoxia-inducible factor 1 subunit alpha (HIF-1*α*) is a member of the key proteins to regulate glycolysis [14]. A range of enzymes involved in glucose uptake and glycolysis are transcriptionally regulated by HIF-1*α*, such as hexokinase 2 (HK2), pyruvate kinase M2 (PKM2), phosphoglycerate kinase 1 (PGK1), and lactate dehydrogenase A (LDHA) [15]. The regulation of HIF-1*α* involves a complex and tightly controlled mechanism. Previous theories suggested that HIF-1*α* is continuously produced but quickly degraded through a proteasome-dependent mechanism [16]. In this process, HIF-1*α* is hydroxylated by specific prolyl hydroxylase domain proteins (PHDs), and the hydroxylated HIF-1*α* is then recognized by von Hippel–Lindau (VHL) for degradation in the proteasome [17, 18]. However, hydroxylation of HIF-1*α* is inhibited under hypoxic conditions, allowing it to accumulate and translocate to the nucleus. Once inside the nucleus, stabilized HIF-1*α* forms a complex with its heterodimer HIF-1*β*, and then binds to specific DNA sequences known as hypoxia-response elements (HREs) in the promoter regions of target genes.

Chaperone-mediated autophagy (CMA) is a discriminative lysosomal-centric protein degradation pathway that involves the recognition of substrates by the cytosolic chaperone heat shock cognate protein 70 (HSC70), and the subsequent degradation of substrates in the lysosomal compartment [19–21]. During the CMA process, lysosomes play a crucial role by providing the site of degradation for the targeted proteins. Lysosome-associated membrane protein type 2A (LAMP2A) is a lysosomal membrane protein that acts as a receptor for the recognition and translocation of target proteins into the lysosome. Once inside the lysosome, the target protein is rapidly degraded by lysosomal proteases, such as cathepsins, resulting in its complete degradation [22, 23]. Approximately 30% of cytosolic proteins carrying the “KFERQ” motif can be degraded by CMA, including HIF-1*α* [24], c-MYC [25], TFEB [26], and several glycolytic enzymes [27–29].

The lysosomal membrane contains several highly glycosylated membrane proteins, with lysosome-associated membrane proteins 1 and 2 (LAMP1/2) accounting for a major portion of the lysosomal membrane glycoproteins [30]. The oligosaccharides present in these proteins contribute significantly to the lysosomal glycocalyx, which protects the membrane components of the lysosome from degradation by hydrolytic enzymes [31]. Among various types of glycosylation, *N-*glycosylation is highly conserved in lysosomes [32]. The oligosaccharyltransferase (OST) multimeric complex resides within the endoplasmic reticulum (ER), where it facilitates the transfer of preassembled oligosaccharides to specific asparagine residues for the generation of *N-*glycosylation [33]. There are two distinct OST complexes in mammals, OST-A and OST-B, that share a series of non*-*catalytic subunits (ribophorin*-*I/II, OST48, DAD1, TMEM258, and OST4) [34]. OST48 and DAD1 are reported to be essential for maintaining the stability of the entire OST complex [35, 36]. Recent studies have demonstrated that abnormal glycosylation of LAMP1 may contribute to Niemann–Pick disease, type C (NPC) pathology [37]. Besides, changes in the electrophoretic mobility of lysosome-associated membrane proteins (LAMPs) were observed in an induced pluripotent stem cell-derived cardiomyocyte (iPSC-CM) model of infantile-onset Pompe cardiomyopathy [38]. Nonetheless, the comprehensive effects of glycosylation modifications in LAMPs on lysosomal function remain to be systematically explored.

In the present study, we identified that ALKBH1 promotes the glycolysis process by inhibiting CMA-mediated degradation of HIF-1*α* in a demethylase-independent manner. Mechanistically, knockdown of ALKBH1 enhances CMA activity and increases the interaction of HIF-1*α* and LAMP2A. Furthermore, we identified that ALKBH1 silencing led to a robust interaction between OST48 and STT3A or DAD1, resulting in the glycosylation of LAMPs, eventually enhancing the function of lysosome and CMA activity. Taken together, our results demonstrate that ALKBH1 competitively binds to OST48, which destroyed the structural integrity among subunits of the OST complex, resulting in the glycosylation disorders of LAMPs and dysfunction of lysosomes, eventually inhibiting the CMA-mediated degradation of HIF-1*α* and promoting glycolysis.

## Materials and methods

### Cell culture

293 T (SCSP-502), HeLa (SCSP-504), SH-SY5Y (SCSP-5014), and U-2 OS (SCSP-5030) cells were obtained from the Shanghai Cell Bank, Type Culture Collection Committee, Chinese Academy of Sciences. ATG7 KO of HeLa cells was a gift from Zhiying Song, Wuhan University. All the cells were cultured in Dulbecco’s Modified Eagle Medium (DMEM) (11995065, Gibico) containing 10% fetal bovine serum and 1% penicillin and streptomycin (15140–122, Gibico) at 37 °C under 5% CO_2_ conditions.

### Plasmids

For the shRNA design of ALKBH1 and OST48, the WI siRNA Selection Program (https://sirna.wi.mit.edu/) was utilized to identify optimal shRNA targets. The resulting target sequences are provided below:

$$\begin{gathered} {\text{shALKBH1}} - {1}:{ 5}^{\prime} - {\text{AAGGTGATCAAATCTCAGCTA}} - {3}^{\prime} ; \hfill \\ {\text{shALKBH1}} - {2}:{ 5}^{\prime} - {\text{GACCGTAGGCTACCATTATAA}} - {3}^{\prime} ; \hfill \\ {\text{shOST48}}:{ 5}^{\prime} - {\text{GAGGACCTTCCTGAAGAAGAA}} - {3}^{\prime} . \hfill \\ \end{gathered}$$

The shRNA sequences designed to target LAMP2A: 5′-GACCGTAGGCTACCATTAT AA-3′ were derived from a study conducted by Igor Tokarchuk et al. [39]. All the target sequences for shRNA were cloned into pLKO.1 vector (Addgene plasmid #10878) according to the provided instructions. For overexpression plasmid, the coding sequence (CDS) of human ALKBH1, along with its enzyme activity center mutant (D233A), was cloned into a modified pSIN-hSNCA-NE vector. The open reading frames (ORFs) of OST48, HIF-1*α*, and LAMP2A were amplified from cDNAs of 293 T cells and subsequently cloned into either the pK-Myc or pLVX-Puro vector. The pSIN-PAmCherry-KFERQ-NE was a gift from Shu Leong Ho (Addgene plasmid # 102365)[40].

### Lentivirus package and transfection

In brief, specific plasmids were transfected into 293 T cells along with psPAX2 and pMD2.G using polyethylenimine (PEI) transfection reagent (40816ES02, YEASEN). Viral supernatants were, respectively, collected at 48 and 72 h post-transfection and utilized to infect target cells in the presence of 10 μg/mL polybrene. After 24 h, the medium was supplemented with 1-2 μg/mL puromycin to select for stably infected cells.

### Western blot analysis

The cells were lysed using ice-cold RIPA buffer (P0013B, Beyotime) supplemented with 1% protease inhibitor cocktail (B14002, Selleck) and 1 mM PMSF (ST506, Beyotime). Following the determination of protein concentrations through the bicinchoninic acid assay (BCA), the cell lysates were combined with 4 × SDS-loading buffer and subjected to boiling at 95 °C for 10 min. Each protein sample (10–30 μg) was loaded and fractionated by SDS-PAGE gels and subsequently transferred onto PVDF membranes. The membranes were then blocked for 1 h with 5% skim milk, followed by incubation with specific primary and HRP-conjugated secondary antibodies (Supplementary Table 1). Immunoreactive bands were visualized using chemiluminescence detection reagents (P0018, Beyotime), and the intensities of the bands were quantified by Quantity One 1D Analysis Software (Bio*-*Rad, CA).

### Quantitative real-time PCR

Total RNA was isolated using the RNAiso Plus reagent (#9109, TaKaRa) by the manufacturer’s protocol. Subsequently, one microgram of the purified total RNA was reverse transcribed into cDNA using the ABScript II cDNA First Strand Synthesis Kit (RK20400, ABclonal). The relative expression of diverse genes was quantified by real-time PCR employing the SYBR Green Master Mix (RK21203, ABclonal) on a CFX96 real-time detection system (Bio-Rad, Hercules, CA). Actin beta (ACTB) was employed as an internal control for normalization, and the results were determined using the 2^−ΔΔCt^ method. Primer sequences for target genes are shown in Supplementary Table 2.

### Immunofluorescence

The cells were fixed with 4% paraformaldehyde for 15 min after reaching 60% confluence on coverslips. The fixed coverslips were rinsed three times with PBS and permeabilized with 0.2% Triton X-100 for 10 min. Afterward, the cells were blocked with 5% bovine serum albumin. The blocked cells were incubated with primary antibodies overnight at 4 °C followed by the secondary antibodies for 1 h protected from light at room temperature (Supplementary Table 1). Cell nuclei were visualized by counterstaining with 4’,6-diamidino*-*2phenylindole (DAPI). After rinsing with PBS, the cells were mounted with an anti-fluorescent quenching sealing agent (P0128S, Beyotime) and imaged using a Leica TCS SP8 confocal microscope.

### Measurement of CMA activity

The activity of CMA was determined using a previously described fluorescent reporter, PAmCherry-KFERQ, composed of a photoactivable mCherry fluorescent protein fused to a KFERQ classic motif [40]. To establish CMA reporter expression, cells were infected with lentiviral particles encoding pSIN-PAmCherry-KFERQ-NE and subsequently selected with puromycin. Monoclonal cell strains stably expressing the PAmCherry-KFERQ were isolated via the limited dilution method. Afterward, gene deletion or expression was performed within the cell strain, followed by seeding in 12-well plates with microscope-cover glasses. Before measurement, the modified cells were photoactivated with a 405 nm light-emitting diode LED for 5 min, followed by serum deprivation for 12 or 18 h. After being fixed with 4% paraformaldehyde, confocal microscopy was used to quantify the number of red foci, which are indicative of CMA activity.

### Co*-*immunoprecipitation and mass spectrometry

293 T cells were transfected with indicated plasmids for 48 h before lysed with western/IP buffer containing protease inhibitors (P0013, Beyotime) and sonicated. The lysates were then centrifuged at 12,000 × *g* for 15 min, and the protein concentrations of the supernatant were determined using the BCA Protein Assay Kit (MA0082, meilunbio). To minimize non*-*specific binding, the lysates underwent preclearance with control protein A/G beads for 4–6 h before incubating with immunoglobulin G (IgG) or specific antibody conjunction beads at 4 °C overnight (Supplementary Table 1). Following the formation of the protein*-*beads complex, the complex was washed three times with prechilled wash buffer. The resulting precipitates were boiled with 1 × SDS buffer for 10 min and loaded onto SDS-PAGE gels for subsequent western blot analysis. For mass spectrometry analysis, immunoprecipitated proteins were separated by SDS-PAGE and stained with Coomassie Brilliant Blue R-250. The visible protein bands were excised and sequentially reduced with tris (2-carboxyethyl) phosphine hydrochloride and alkylated with chloroacetamide and, following trypsin or chymotrypsin digestion, were subjected to HPLC–MS analysis. Data analysis was performed by Fitgene Biotech Co. (Guangzhou, China), and the identified proteins were further validated through Co*-*IP experiments.

### Electron microscopy

The procedure for transmission electron microscopy (TEM) was conducted following a previously described method [41]. Briefly, 1 × 10^7^ cells were initially collected through pancreatic digestion and subsequently fixed with 2.5% glutaraldehyde in 0.01 M phosphate buffer (G1102, Servicebio) at room temperature protected from light for 30 min. Subsequently, the cell samples were rinsed in 100 mM cacodylate buffer and post-fixed in 1% osmium tetroxide for an additional 1 h at 4 °C. Thereafter, the fixed samples were embedded in EMBed 812 epoxy resin (90529-77-4, SPI) following dehydrated using an ethanol gradient. Ultrathin Sects. (60 nm) were then collected on copper grids and stained with 2% uranyl acetate in saturated alcohol for 8 min, followed by exposure to 2.6% lead citrate for 8 min. The resulting images were captured using a Hitachi HT7800 transmission electron microscope.

### Measurement of pyruvic acid and lactate level

The intracellular content of pyruvate, as well as lactate levels in the cell supernatant, were assessed using the Pyruvate Assay Kit (BC2205, Solarbio) and Lactate Assay Kit (BC2230, Solarbio) following the manufacturer’s recommended protocol. Briefly, cells from 6-well plates were collected into centrifuge tubes, and commercial extracts were added before sonication. Following centrifugation, the resulting supernatant was collected and used for subsequent analysis. For lactic acid, the cell supernatant was collected after 4 h of incubation for subsequent analysis. The absorbance value of the sample was then substituted into the generated standard curve to determine the corresponding index measurement. All obtained data were further normalized to the corresponding protein concentration of the cell extracts.

### Measurement of glycolytic capacity

The extracellular acidification rate (ECAR) was analyzed by the Seahorse Bioscience XF96 Extracellular Flux Analyzer in conjunction with the Seahorse XF Glycolysis Stress Test Kit (103020–100, Agilent). Briefly, cells (1.5 × 10^4^/well) were seeded into 96-well Seahorse XF cell culture microplates and allowed to adhere overnight. Subsequently, cells were incubated with XF Base Medium containing 2 mM glutamine, followed by sequential injection of 10 mM glucose, 1.5 μM oligomycin, and 50 mM 2-deoxyglucose (2-DG). The acquired data were further normalized to the corresponding protein concentration of the cell extracts.

### Subcellular fractionation

The cytoplasmic and nuclear fractions were separated using a Nuclear and Cytoplasmic Protein Extraction Kit (P0027, Beyotime) following the manufacturer’s protocols. Briefly, cells were washed with cold PBS and harvested by centrifugation. Subsequently, cells were lysed with cytosolic extraction buffer supplemented with protease inhibitors (P0013, Beyotime) and kept on ice for 10 min after brief vortexing. The lysates were centrifuged at 12,000 × *g* for 5 min, and the resulting supernatants were collected as the cytoplasmic fraction. The pellets were subsequently washed three times with cytosol extraction buffer and lysed with nuclear extraction buffer containing protease inhibitors. After incubation on ice for 30 min, the nuclear fraction was obtained by centrifugation at 12,000 × *g* for 10 min. The protein concentration of each fraction was determined using the BCA Protein Assay Kit (MA0082, meilunbio), and then subjected to immunoblotting.

### Statistics and reproducibility

All statistical analyses were performed using Excel (Microsoft) or Prism (GraphPad 8.0) software. The statistical results are presented as the mean ± standard deviation (S.D.), and *p* values were calculated using unpaired two*-*tailed Student’s *t* test for pairwise comparisons. *p* < 0.05 was considered as statistical significance (* indicates *p* < 0.05; ** indicates *p* < 0.01; *** indicates *p* < 0.001). All experiments were performed independently at least three times unless stated otherwise in the figure legend.

## Results

### ALKBH1 knockdown suppresses glycolysis via downregulating HIF-1*α*

**Fig. 1:**
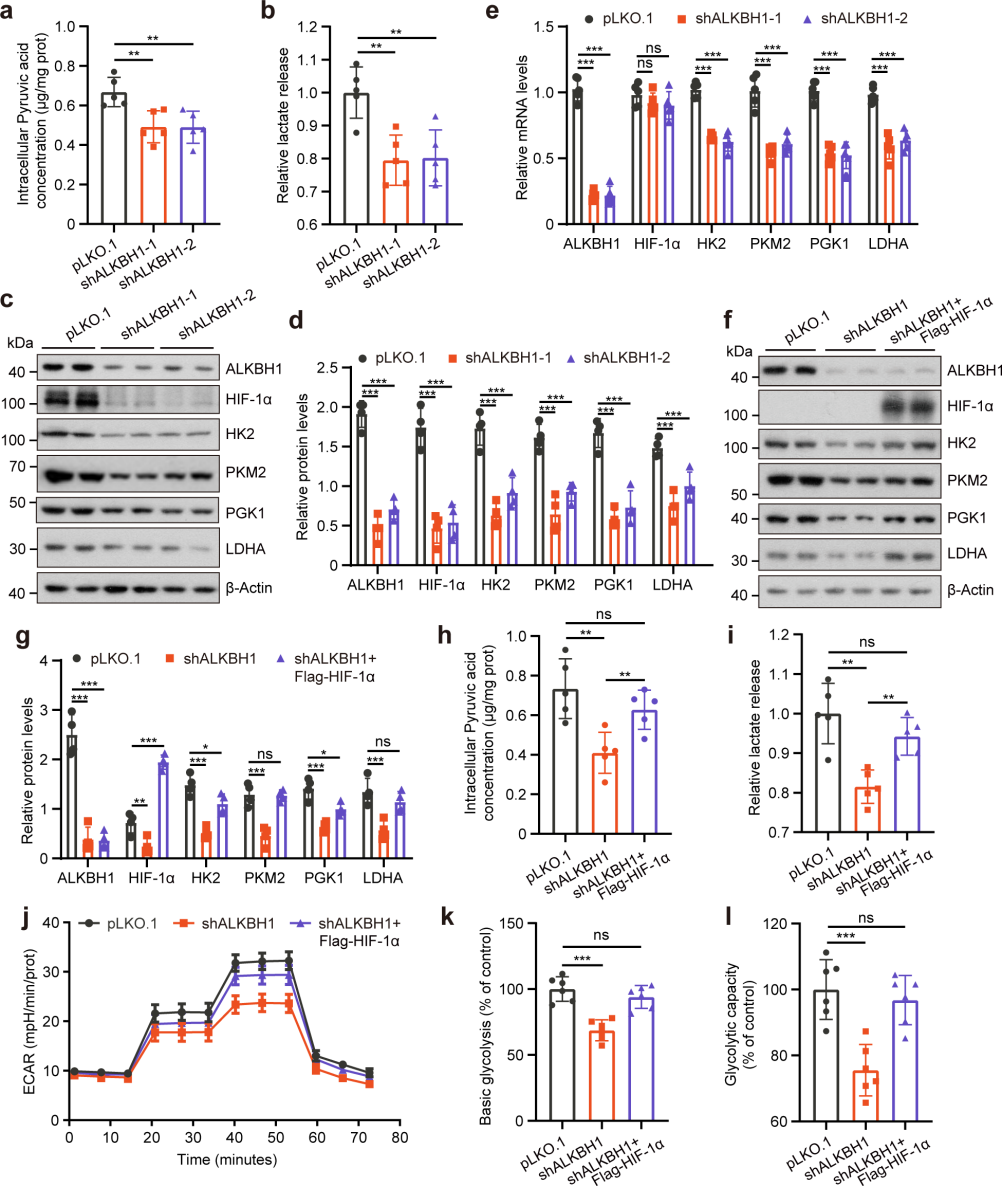
ALKBH1 knockdown suppresses glycolysis via downregulating HIF-1*α*. **a**, **b** Intracellular pyruvic acid concentration (**a**) and lactate release (**b**) were measured in control and ALKBH1 knockdown HeLa cells, relative data were further normalized to the control cells (*n* = 5 independent samples). **c**, **d** Western blots (**c**) and quantitative results (**d**) of the indicated proteins in control and ALKBH1 knockdown HeLa cells (*n* = 4 independent samples). **e** Relative transcript levels of the indicated gene were analyzed by q-PCR in control and ALKBH1 knockdown HeLa cells, ACTB was used as a control (*n* = 5 independent samples). **f**, **g** Western blots (**f**) and quantitative results (**g**) of the indicated proteins in control and ALKBH1 knockdown cells transfected with Flag-tagged HIF-1*α* (*n* = 4 independent samples). **h**, **i** Intracellular pyruvic acid concentration (**h**) and lactate release (**i**) were measured in control, ALKBH1 knockdown, and ALKBH1 knockdown cells transfected with Flag-tagged HIF-1*α* (*n* = 5 independent samples). **j–l** Extracellular acidification rate (ECAR) (**j**), basic glycolysis (**k**), and glycolytic capacity (**l**) were detected in control, ALKBH1 knockdown, and ALKBH1 knockdown cells transfected with Flag-tagged HIF-1*α* at different time points (*n* = 6 independent samples). Data are presented as mean values ± SD; *p < 0.05, **p < 0.001, ***p < 0.001; ns indicated no significance

Previous research has demonstrated that ALKBH1 is involved in the demethylation of 6 mA of HIF-1*α* and GYS1, leading to enhanced activation of the HIF-1 pathway during adipogenic differentiation [9]. Since HIF-1*α* is a central regulator of glycolysis processes, we wonder whether ALKBH1 plays a role in regulating glycolysis through HIF-1*α*. To address this question, we analyzed the RNA-seq results of ALKBH1 knockout and control cells from the GEO database and identified that both hypoxia and glycolysis-related pathways are significantly decreased in the ALKBH1 knockout group (Supplementary Fig. 1a), suggesting a potential role for ALKBH1 in regulating glycolysis through HIF-1*α*. To validate these results, we initially performed loss-of-function assays by pLKO.1-shRNA-mediated knockdown system and quantified intracellular pyruvate and lactate production in HeLa cells. Compared with cells expressing empty shRNA, reduced ALKBH1 expression resulted in decreased pyruvate content and lactate production (Fig. 1a, b). Furthermore, ALKBH1 knockdown reduced the mRNA and protein levels of the key enzymes of the glycolysis pathway, including HK2, PKM2, PGK1, and LDHA. Remarkably, knockdown or overexpression of ALKBH1 did not affect the mRNA level of HIF-1*α*, although it had a severe effect on the protein level of HIF-1*α* (Fig. 1c–e; Supplementary Fig. 1b). To further confirm that HIF-1*α* is responsible for ALKBH1 mediated glycolysis, we reintroduced HIF-1*α* in ALKBH1 knockdown cells. Ectopic expression of HIF-1*α* restored ALKBH1 knockdown*-*mediated reduction of glycolysis key enzymes and glycolytic intermediate (Fig. 1f–i). Subsequently, we examined the glycolytic flux of ALKBH1 knockdown and control cells by directly measuring the extracellular acidification rate (ECAR). We detected a reduction in the level of basic glycolysis and glycolytic capacity with ALKBH1 depletion, which was significantly reversed by the expression of HIF-1*α* (Fig. 1j–l). In addition, the reintroduction of ALKBH1 via plasmid transfection in knockdown cells effectively restored the expression of glycolysis-related genes (Supplementary Fig. 1c, d). These data collectively suggest that ALKBH1 is evidently involved in the regulation of glycolytic metabolism which is achieved through HIF-1*α*.

### Depletion of ALKBH1 promotes the lysosomal degradation of HIF-1*α*

**Fig. 2:**
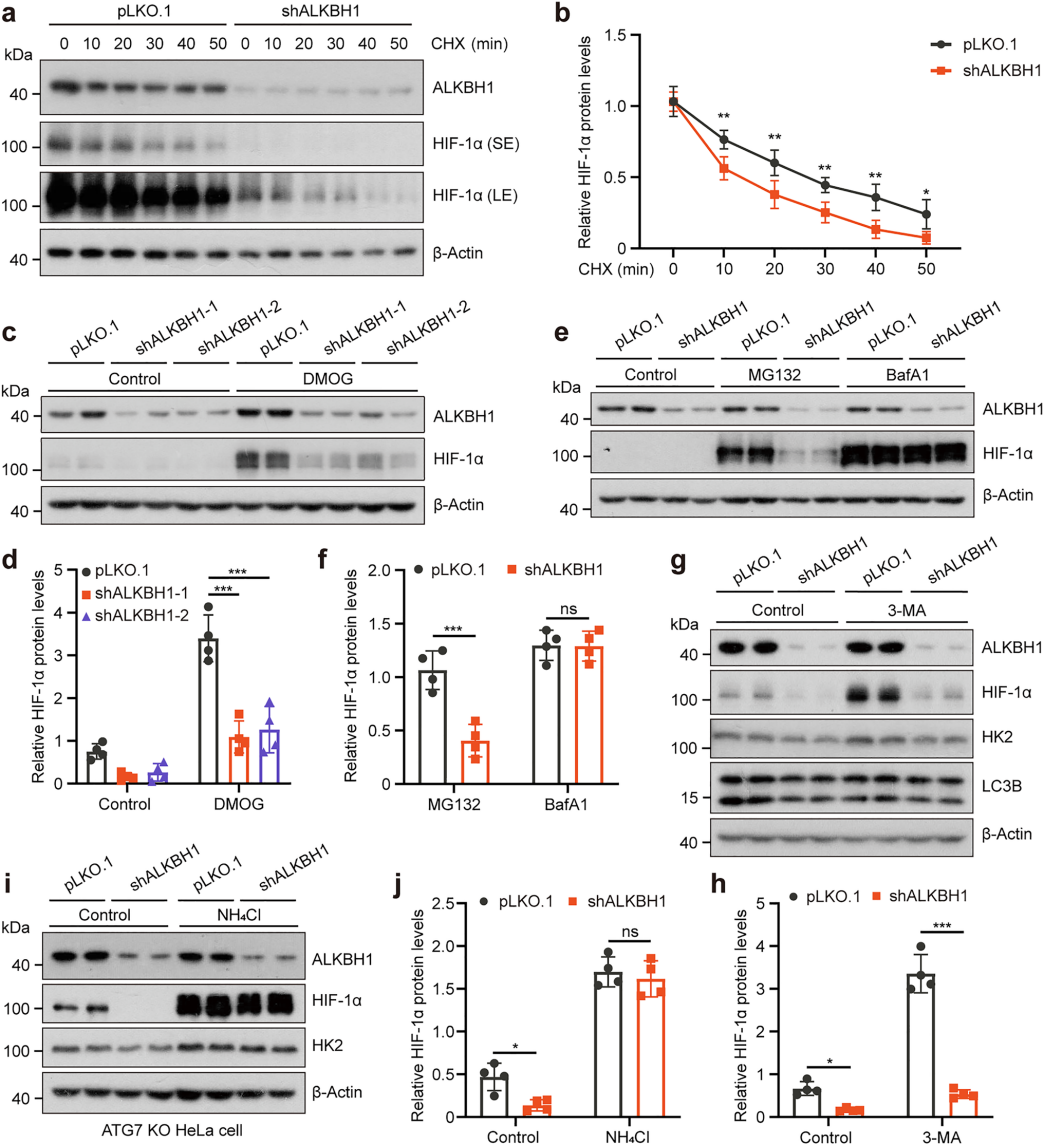
Depletion of ALKBH1 promotes the lysosomal degradation of HIF-1*α*. **a, b** Western blots (**a**) and quantitative results (**b**) indicated the protein level of HIF-1*α* between control and ALKBH1 knockdown of HeLa cells treated with 10 μM cycloheximide (CHX) for different times (*n* = 4 biological replicates). **c, d** Western blots (**c**) and quantitative results (**d**) demonstrated the HIF-1*α* protein expression in control and ALKBH1 knockdown HeLa cells treated with 100 μM dimethyloxallyl glycine (DMOG) for 12 h (*n* = 4 biological replicates). **e**, **f** Western blots (**e**) and quantitative results (**f**) indicated the HIF-1*α* protein expression in control and ALKBH1 knockdown HeLa cells treated with 10 μM Z-Leu-Leu-Leu-al (MG132) and 100 nM Bafilomycin A1 (BafA1) for 12 h (*n* = 4 biological replicates). **g**, **h** Western blots (**g**) and quantitative results (**h**) of the indicated protein expression after inhibiting the macroautophagy function of control and ALKBH1 knockdown HeLa cells by 5 mM 3-Methyladenine (3-MA), LC3B is used to represent the validity of 3-MA (*n* = 4 biological replicates). **i**, **j** Western blots (**i**) and corresponding quantitative results (**j**) of the indicated proteins in control and ALKBH1 knockdown HeLa ATG7 ko cells after treatment with 20 mM NH_4_CL (*n* = 4 biological replicates). Data are presented as mean values ± SD; *p < 0.05, **p < 0.001, ***p < 0.001; ns indicated no significance

In recent years, the degradation mechanisms of HIF-1*α* have been extensively studied. To explore the potential mechanism by which ALKBH1 regulates the protein levels of HIF-1*α*, a cycloheximide (CHX, an inhibitor of protein synthesis) chase assay was introduced to measure the protein degradation of HIF-1*α* [42]. We observed a significant enhancement in the degradation rate of HIF-1*α* protein in ALKBH1 knockdown cells after CHX treatment (Fig. 2a, b), indicating that ALKBH1 silencing accelerated the degradation of HIF-1*α*. To demonstrate whether HIF-1*α* was degraded through the proteasome pathway, prolyl hydroxylase inhibitor dimethyloxalylglycine (DMOG) was used to block the ubiquitination of HIF-1*α* [43]. However, ALKBH1 deficiency consistently decreased the expression of HIF-1*α* in the presence of DMOG (Fig. 2c, d). Moreover, wild-type cells treated with a PHD inhibitor CoCl_2_, which was proven to be an effective hypoxic mimetic [44], could significantly maintain HIF-1*α* stability. By contrast, ALKBH1-knockdown cells with the same CoCl_2_ treatment were unable to keep the protein level of HIF-1*α* (Supplementary Fig. 2a, b).

There are two primary mechanisms of protein degradation in cells: the ubiquitin*–*proteasome system (UPS) and the autophagy-lysosome pathway (ALP) [45]. To determine whether the effect of ALKBH1 is independent of the proteasome, we analyzed the level of HIF-1*α* in the presence of the UPS inhibitor MG132 or ALP inhibitor Bafilomycin (BafA1). The results indicated that HIF-1*α* expression was restored after treatment with BafA1, but not MG132 (Fig. 2e, f). Similarly, we also observed a significant restoration of HIF-1*α* in 293 T cells in the presence of BafA1 (Supplementary Fig. 2c, d). Furthermore, BafA1 was employed to inhibit the ALP under CoCl_2_ treatment conditions, immunoblot analysis showed that the protein level of HIF-1*α* was restored only in the presence of ALP inhibition (Supplementary Fig. 2e, f). These findings above indicate that depletion of ALKBH1 might promote HIF-1*α* degradation in an autophagy-dependent manner.

According to the mechanism used to deliver cargo to the lysosome, autophagy can be categorized as macroautophagy, microautophagy and CMA [46]. To decipher whether macroautophagy is the mechanism involved in HIF-1*α* degradation, classical markers of macroautophagy were checked in HeLa cells [47]. Immunoblot analysis revealed reduced LC3B-II/LC3B-I ratios and elevated p62 protein expression in ALKBH1 knockdown cells (Supplementary Fig. 3a, b). By contrast, overexpression of ALKBH1 and its demethylase-dead mutation (D233A) both increased the ratio of LC3B-II/I but failed to make a reduction in p62 (Supplementary Fig. 3c, d). In addition, an autophagy reporter expressing the GFP-LC3 fusion protein was employed to assess the activation state of autophagy [48]. The number of autophagosomes GFP-LC3 puncta was decreased in ALKBH1 knockdown cells under basal level and BafA1 treatment conditions (Supplementary Fig. 3e, f). Furthermore, the selective macroautophagy inhibitor 3-methyladenine (3-MA) treatment led to a modest increase in HIF-1*α* expression, but it failed to stabilize HIF-1*α* in ALKBH1 knockdown cells (Fig. 2g, h). We then examined the levels of HIF-1*α* in HeLa cells with an ATG7 knockout configuration, wherein macroautophagy was inhibited as previously described [49]. As anticipated, a persistent reduction in HIF-1*α* was observed in the ATG7 KO cells with ALKBH1 knockdown. However, the level of HIF-1*α* was significantly rescued in the presence of the lysosomotropic neutralizing agent ammonium chloride (NH_4_Cl) (Fig. 2i, j). Collectively, these findings suggest that ALKBH1 depletion*-*mediated degradation of HIF-1*α* is potentially reliant on lysosome pathways, but not the macroautophagy.

### ALKBH1 silencing enhances CMA-mediated degradation of HIF-1*α*

**Fig. 3:**
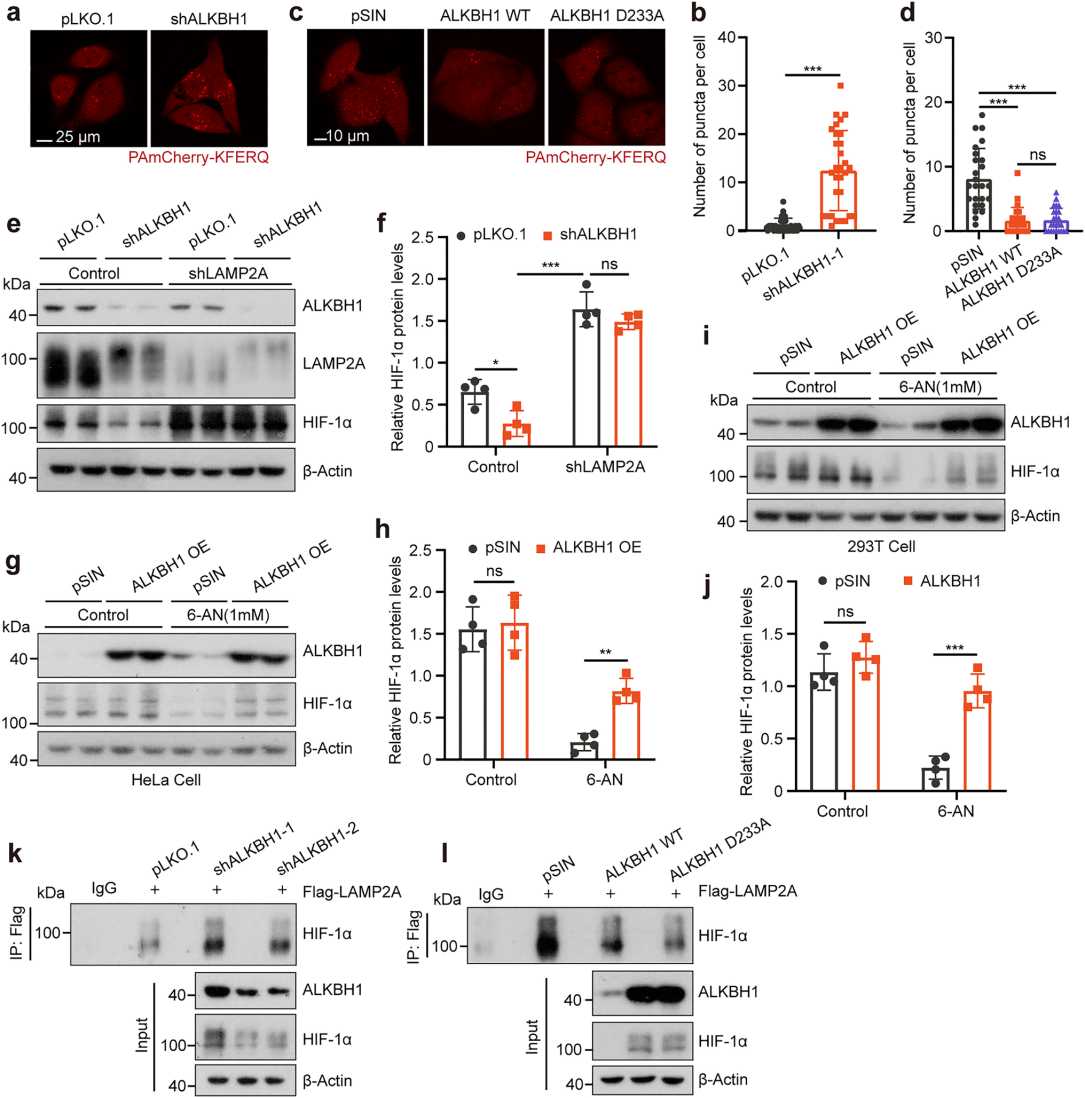
ALKBH1 silencing enhances CMA-mediated degradation of HIF-1*α*. **a, b** Representative images (**a**) and quantitative results (**b**) of PAmCherry puncta (red) in control and ALKBH1 knockdown HeLa cells stably expressing PAmCherry-KFERQ-NE after serum deprivation for 12 h (Scale bar = 25 μm; *n* = 30 independent cells). **c, d** Representative images (**c**) and quantitative results (**d**) of PAmCherry puncta (red) in control, ALKBH1 WT or ALKBH1 D233A overexpression HeLa cells stably expressing PAmCherry-KFERQ-NE after serum deprivation for 18 h (Scale bar = 10 μm; *n* = 25 independent cells). **e, f** Western blots (**e**) and quantitative results (**f**) indicated the HIF-1*α* protein expression in control, ALKBH1 knockdown or ALKBH1, LAMP2A double knockdown HeLa cells after serum deprivation for 12 h (*n* = 4 biological replicates). **g, h** Western blots (**g**) and quantitative results (**h**) indicated the HIF-1*α* protein expression in control and ALKBH1 overexpression HeLa cells with or without 1 mM 6-aminonicotinamide (6-AN) treatment for 12 h (*n* = 4 biological replicates). **i, j** Western blots (**i**) and quantitative results (**j**) of the indicated proteins in control and ALKBH1 overexpression 293 T cells with or without 1 mM 6-AN treatment for 12 h (*n* = 4 independent samples).** k** Immunoprecipitation/immunoblot analysis of the whole-cell lysates (WCLs) and anti-Flag derived from control and ALKBH1 knockdown 293 T cells transfected with Flag-LAMP2A, IgG served as a negative control (*n* = 3 biological replicates). **l** Immunoprecipitation/immunoblot analysis of the WCLs and anti-Flag derived from control, ALKBH1 WT or D233A overexpression 293 T cells transfected with Flag-LAMP2A, IgG served as a negative control (*n* = 3 biological replicates). Data are presented as mean values ± SD; *p < 0.05, **p < 0.001, ***p < 0.001; ns indicated no significance

More recent evidence indicates that the degradation of HIF-1*α* may occur by CMA in an oxygen*-*independent manner [50]. CMA is a lysosomal degradation pathway that eliminates substrate proteins through HSC70 and LAMP2A-assisted translocation [51]. To confirm whether ALKBH1 knockdown*-*mediated degradation of HIF-1*α* is coupled to CMA, a specific fluorescent CMA reporter PAmCherry-KFERQ was used to evaluate CMA activity [40]. Compared with vehicle control, ALKBH1 knockdown cells showed an increase in the number of fluorescent puncta under serum deprivation conditions (Fig. 3a, b). Conversely, overexpression of ALKBH1 WT or D233A lowered the number of fluorescent puncta (Fig. 3c, d), indicating that ALKBH1 might modulate the activity of CMA to influence the HIF-1*α* level. Furthermore, leucine-rich repeat kinase 2 (LRRK2) and myocyte enhancer factor 2D (MEF2D) have been identified as typical CMA substrates in neurodegenerative diseases [52, 53]. Our findings indicated reduced protein levels of LRRK2 and MEF2D in SH-SY5Y cells following ALKBH1 depletion (Supplementary Fig. 3 g, h).

To further validate the role of CMA in HIF-1*α* degradation, the lysosome receptor LAMP2A was silenced by lentivirus-containing shRNA. Depletion of LAMP2A led to a substantial restoration of HIF-1*α* in the ALKBH1 knockdown cells after serum deprivation (Fig. 3e, f). Furthermore, 6-aminonicotinamide (6-AN), a commonly used CMA-stimulating compound, was employed to further validate the effect of ALKBH1 on CMA substrates. Notably, we observed a decrement in the overall level of HIF-1*α* protein following 6-AN treatment, and a slight increase in HIF-1*α* levels when ALKBH1 was overexpressed in both HeLa and 293 T cells, while no change was observed under normal conditions (Fig. 3g–j). As the levels of constitutive CMA activity in most cells are relatively low, it suggests the levels of CMA substrate are unlikely to be altered by ALKBH1 overexpression [54]. Subsequently, the interaction between HIF-1*α* and LAMP2A was assessed through co*-*immunoprecipitation experiments. Immunoblot analysis demonstrated that the amount of HIF-1*α* co*-*precipitated with LAMP2A increased after ALKBH1 depletion, while it was suppressed in cells overexpressing ALKBH1 WT or D233A (Fig. 3k, l). These data corroborate that loss of ALKBH1 promotes HIF-1*α* degradation via enhancing the CMA process.

### ALKBH1 inhibits glycosylation of lysosomal membrane proteins

**Fig. 4:**
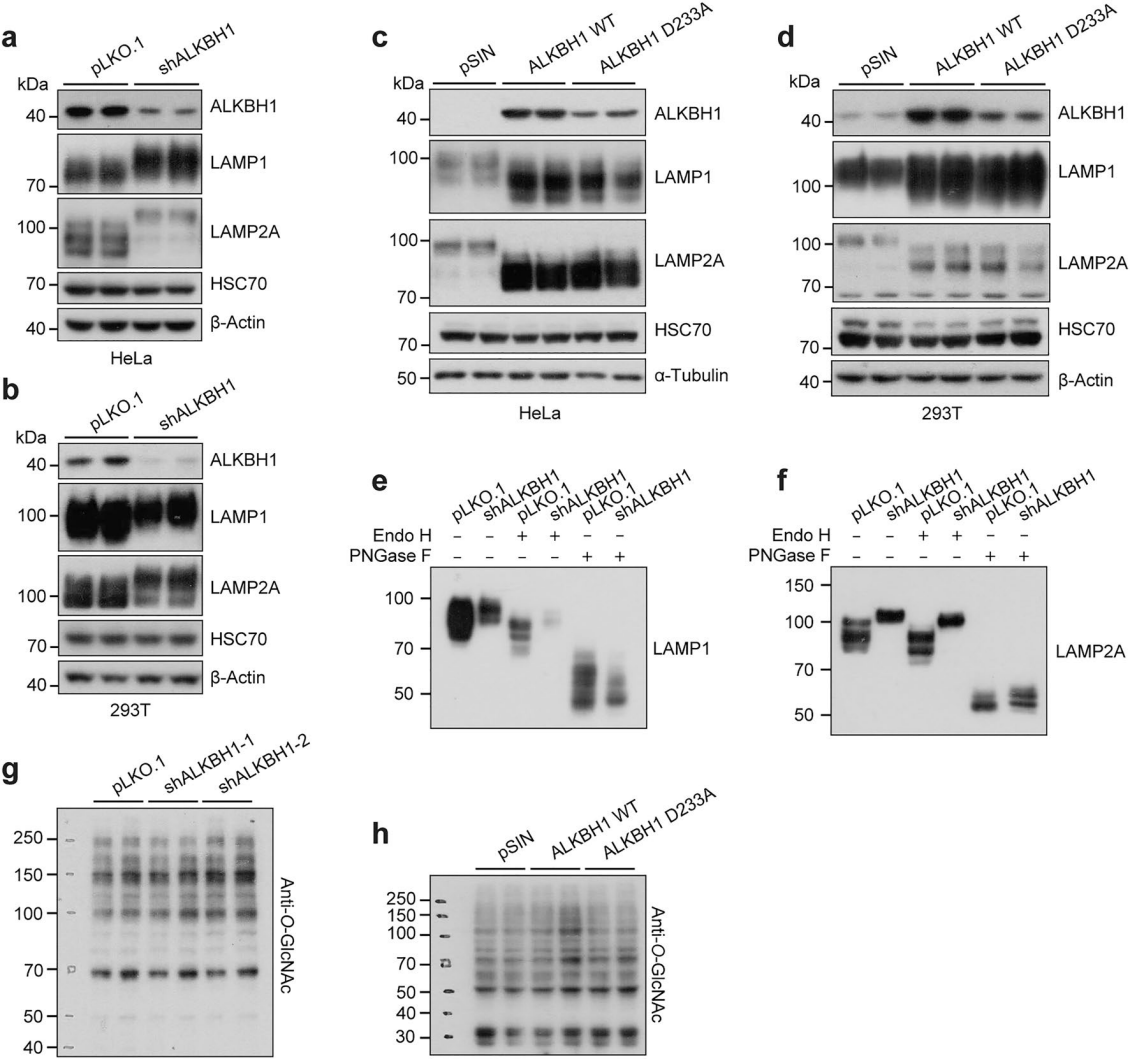
ALKBH1 inhibits glycosylation of lysosomal membrane proteins. **a**, **b** Western blots indicated CMA-associated protein expression in HeLa cells (**a**) and 293 T cells (**b**) after ALKBH1 knockdown (*n* = 3 biological replicates). **c**, **d** Western blots reflected CMA-associated protein expression in control, ALKBH1 WT or ALKBH1 D233A overexpression HeLa cells (**c**) and 293 T cells (**d**) (*n* = 3 biological replicates). **e, f** Western blots indicated the mobility shift of LAMP1 (**e**) and LAMP2A (**f**) after treatment with endoglycosidase H (Endo H) or peptide-*N-*glycosidase F (PNGase F) in ALKBH1 knockdown HeLa cells (*n* = 3 biological replicates). **g** Western blots revealed differential expression of anti-*O-*linked *N-*acetylglucosamine in control and ALKBH1 knockdown HeLa cells (*n* = 3 biological replicates). **h** Western blots revealed differential expression of anti-*O-*linked *N-*acetylglucosamine in control, ALKBH1 WT or ALKBH1 D233A overexpression HeLa cells (*n* = 3 biological replicates)

Intriguingly, we observed a higher band of LAMP2A around 100 kDa in the ALKBH1 knockdown cells (Fig. 3e), which were likely to be the glycosylated LAMP2A. The lysosomal membrane contains several highly glycosylated membrane proteins, LAMP1 and LAMP2A account for a major portion of the lysosomal membrane glycoproteins [30]. We confirmed that both LAMP1 and LAMP2A exhibited a pronounced mobility shift in both HeLa and 293 T cells upon ALKBH1 knockdown (Fig. 4a, b). By contrast, overexpression of either WT or D233A ALKBH1 led to a downshift in the LAMP1/LAMP2A band in both HeLa and 293 T cells (Fig. 4c, d), suggesting that ALKBH1 might cause a hindrance in LAMPs glycosylation. However, the mRNA levels of LAMP1 or LAMP2A did not change in either ALKBH1 knockdown or overexpression cells (Supplementary Fig. 4a, b). To confirm that the observed shifts in mobility were a result of aberrant *N-*glycosylation, endoglycosidase H (Endo H) and peptide-*N-*glycosidase F (PNGase F) digestion assays were performed, which selectively remove immature high mannose-containing sugars and all *N-*linked sugars, respectively [55]. Upon treatment with Endo H, only a slight band shift was observed compared to the control group, with neither LAMP1 nor LAMP2A converging with vector control cells, suggesting they are modified with fewer *N-*linked high mannose-containing or hybrid sugars. Nonetheless, PNGase F caused a significant downshift of protein bands along with the converged mobility of LAMP1/LAMP2A between control and knockdown cells, demonstrating that the observed faster mobility was indeed caused by altered *N-*glycosylation (Fig. 4e, f).

The luminal domains of LAMP1 and LAMP2 are extensively glycosylated, predominantly composed of *N-*linked glycans and *O-*linked glycans [37]. To avoid the misinterpretation of potential *O-*linked glycosylation, *O-*linked *N-*acetylglucosamine (*O-*GlcNAc) was detected using a pan*-*specific antibody for protein modifications. Immunoblot analyses revealed that neither ALKBH1 knockdown nor overexpression altered the *O-*linked modifications in HeLa cells (Fig. 4g, h). These data collectively demonstrate that ALKBH1 inhibits *N-*glycosylation of lysosomal membrane proteins.

### Overexpression of ALKBH1 triggers dysfunction of lysosome

**Fig. 5:**
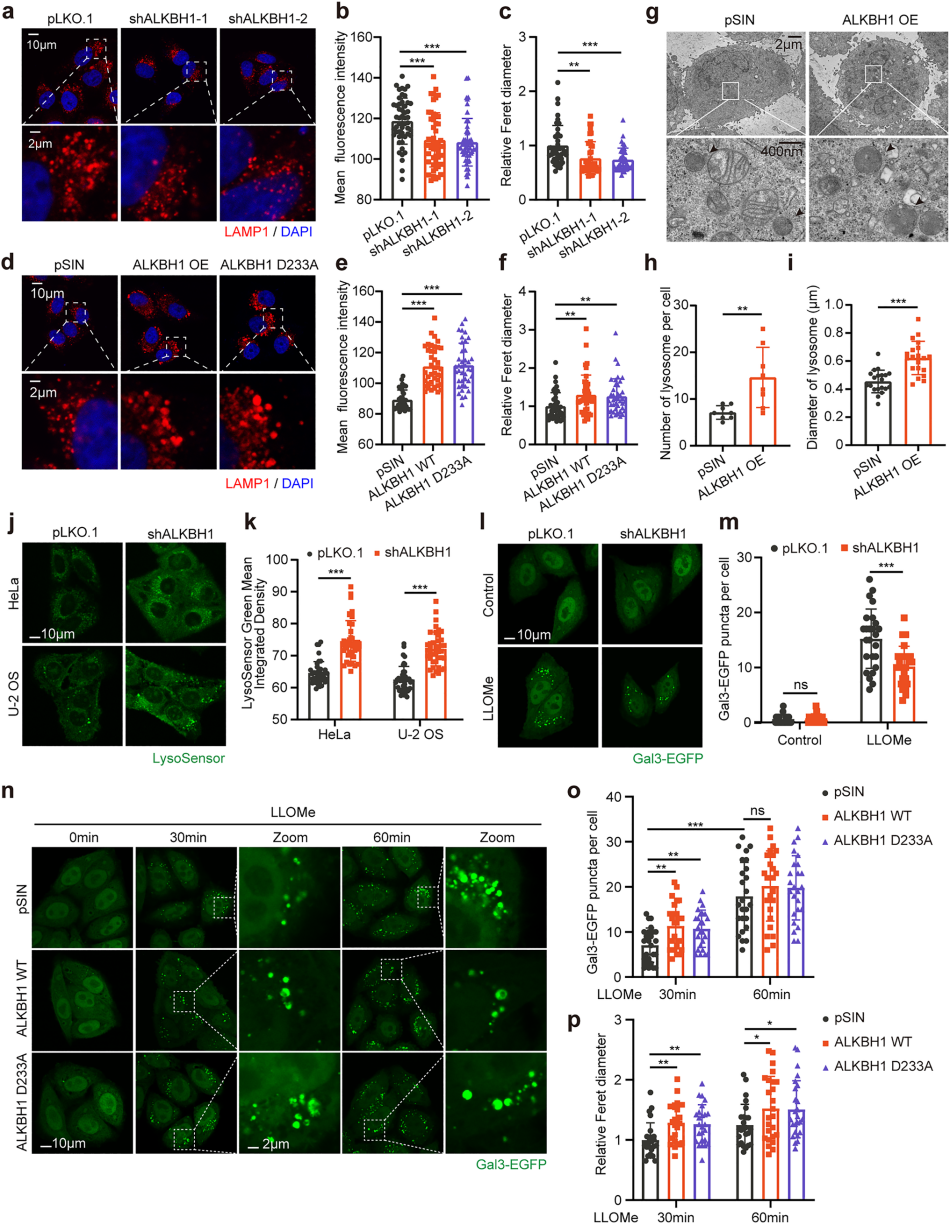
Overexpression of ALKBH1 triggers dysfunction of lysosome. **a–c** Representative images (**a**) and quantitative results indicated the mean fluorescence intensity (**b**) and relative Feret diameter (**c**) of LAMP1 puncta (red) in control and ALKBH1 knockdown HeLa cells (Scale bar = 10 μm, *n* = 50 independent cells; Scale bar = 2 μm by zoomed in, *n* = 40 independent puncta). **d–f** Representative images (**d**) and quantitative results reflect the mean fluorescence intensity (**e**) and relative Feret diameter (**f**) of LAMP1 puncta (red) in control and ALKBH1 WT or D233A overexpression HeLa cells (Scale bar = 10 μm, *n* = 40 independent cells; Scale bar = 2 μm by zoomed in, *n* = 40 independent puncta). **g–i** Representative electron microscopic images (**g**) and quantification reflect the number of lysosomes per cell (**h**) and the diameter of lysosome (**i**) in control and ALKBH1 overexpression HeLa cells (*n* = 8 independent cells in number of lysosomes; *n* = 20 independent fields in diameter of lysosome). **j**, **k** Representative images (**j**) and quantitative results (**k**) indicate the mean integrated density of LysoSensor Green in control and ALKBH1 knockdown HeLa or U-2 OS cells (Scale bar = 10 μm, *n* = 40 for HeLa cells or U-2 OS cells). **l**, **m** Representative images (**l**) and quantitative results (**m**) reveal the number of Gal3-EGFP puncta per cell in control and ALKBH1 knockdown HeLa cells stably expressing Gal3-EGFP after 1 mM LLOMe treated for 60 min. (Scale bar = 10 μm; *n* = 25 independent cells). **n–p** Representative images (**n**) and quantitative results indicate the number of Gal3-EGFP puncta (**o**) and relative Feret diameter (**p**) in control, ALKBH1 WT or D233A overexpression HeLa cells stably expressing Gal3-EGFP under LLOMe treatment for 30 min or 60 min (Scale bar = 10 μm, n = 25 independent cells; Scale bar = 2 μm by zoomed in, *n* = 25 independent puncta). Data are presented as mean values ± SD; *p < 0.05, **p < 0.001, ***p < 0.001; *ns* indicated no significance

The highly glycosylated lysosome-associated membrane proteins (LAMPs) could protect the lysosomal membrane from degradation, maintain ion homeostasis, and transport cholesterol and other lipid [56]. Therefore, we hypothesized that ALKBH1 knockdown may regulate the function of lysosomes. We first assessed the number of lysosomes by lysosome-specific fluorescence staining. Immunofluorescence images showed that the mean fluorescence intensity (MFI) of LAMP1-positive puncta decreased in ALKBH1 knockdown cells (Fig. 5a, b), while a higher MFI of LAMP1 per cell was observed following ALKBH1 overexpression (Fig. 5d, e). In addition, we observed that the volume of lysosomes labeled with LAMP1 was smaller in knockdown cells upon closer examination of the fluorescent images (Fig. 5a, c). Conversely, the lysosomes appeared enlarged in cells overexpressing ALKBH1 compared to the control groups (Fig. 5d, f). Moreover, transmission electron microscopy (TEM) was employed to capture images of lysosomes. Compared to the control cells, the ultrastructure of ALKBH1 overexpressed cells revealed a significant increase in the number of intracellular lysosomes, as well as an expansion in lysosomal volume (Fig. 5g–i).

As the cellular degradation and recycling center, lysosomes house over 60 different types of hydrolases and maintain an acidic environment within the membrane, with a pH ranging between 4.5 and 5.5 [57]. Abnormal fluctuations in lysosomal pH can lead to lysosomal dysfunction and consequently result in the accumulation of enlarged autophagic vesicles [58]. Therefore, LysoSensor green DND-189, a pH-sensitive dye that labels and tracks lysosomes, was used to visualize lysosomal pH. Immunofluorescence images demonstrated that ALKBH1 silencing led to intensified fluorescence of LysoSensor Green both in HeLa and U-2 OS cells (Fig. 5j, k). In addition, the mean integrated density of DND-189 was slightly decreased in ALKBH1 WT and D233A-overexpressing cells, indicating higher lysosomal pH (Supplementary Fig. 5a, b).

Lysosomes are single-membrane cell organelles, multiple endogenous and exogenous stimuli can lead to lysosomal membrane permeabilization (LMP) [59]. Severe LMP caused the release of lysosomal hydrolases, which triggered cell death [60]. Previous studies have shown that induction of LMP enables the immediate translocation of Galectin 3/LGALS3 from the cytosol to the lysosomal lumen, making it a visualized marker of LMP [61]. To further investigate whether ALKBH1 has an impact on LMP, we initially assessed the fluorescence granules of endogenous Galectin 3 in ALKBH1-depleted cells. Immunofluorescence images revealed that ALKBH1 silencing resulted in a decreased count of Galectin 3 puncta in HeLa cells following treatment with LLOMe (Supplementary Fig. 5c, d). To intuitively observe the situation of LMP, a fluorescent reporter stably expressing the Galectin 3-EGFP was established as previously described[62]. The EGFP-tagged Galectin 3 (Gal3-EGFP) puncta that appeared upon LLOMe treatment exhibited a similar decrease when ALKBH1 was suppressed (Fig. 5l, m). By contrast, the number of Gal3-EGFP puncta increased with the overexpression of WT or D233A ALKBH1 (Fig. 5n, o). Intriguingly, upon closer examination of the immunofluorescence images, we observed an enlarged and vacuolated morphology of Galectin 3 aggregation, resembling the lysosomes labeled with LAMP1 in ALKBH1 WT or D233A overexpressed cells (Fig. 5n, p). This phenomenon was also observed in U-2 OS cells (Supplementary Fig. 5e–g). Overall, the overexpression of ALKBH1 resulted in both morphological and functional impairment of the lysosomes.

### ALKBH1 affects *N-*glycosylation by interfering with OST complex

**Fig. 6:**
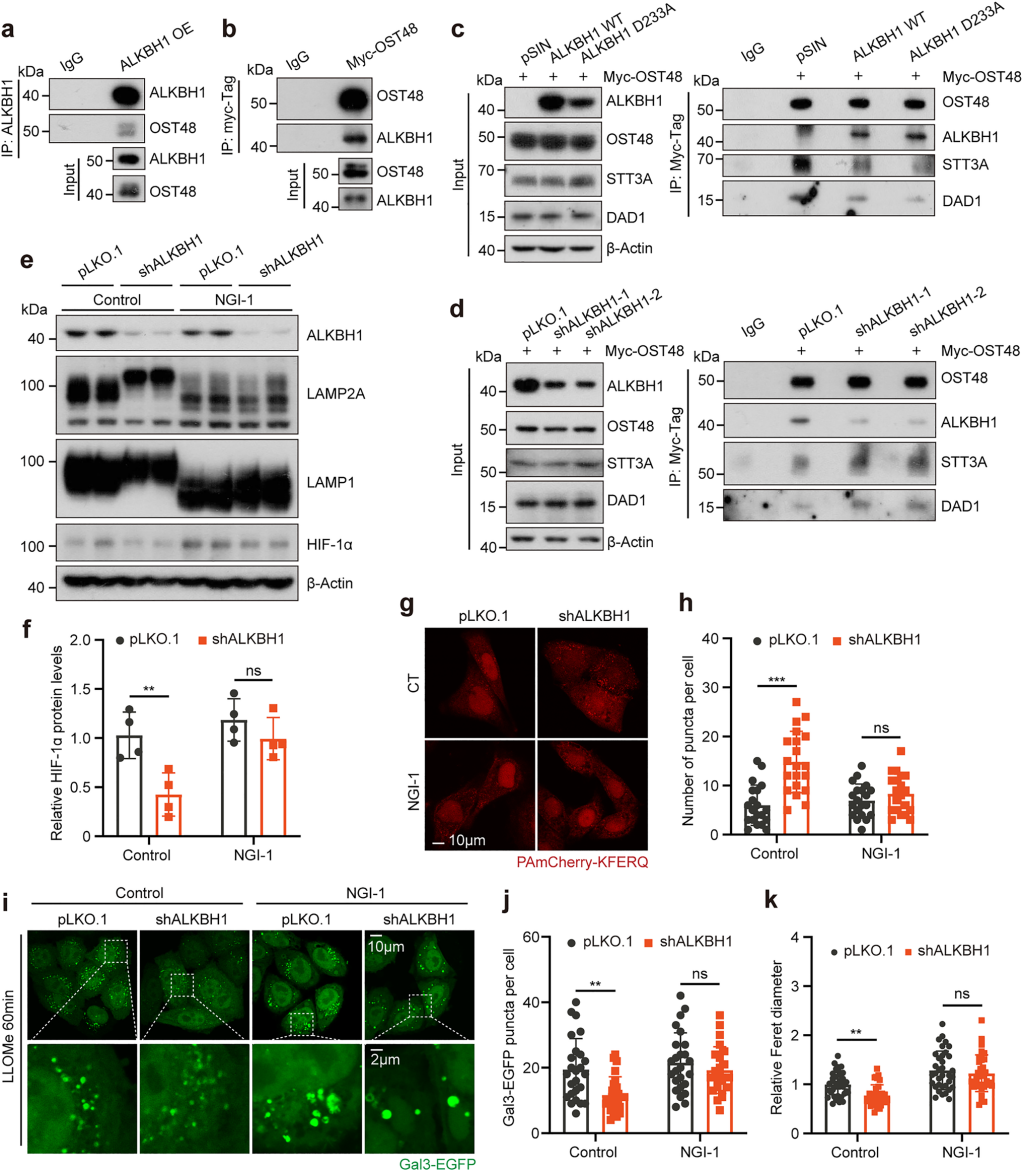
ALKBH1 affects *N-*glycosylation by interfering with OST complex. **a, b** Immunoprecipitation/immunoblot analysis of the WCLs indicated anti-ALKBH1 (**a**) or anti-Myc (**b**) derived from 293 T cells transfected with ALKBH1 or Myc-OST48, IgG served as the negative controls (*n* = 3 biological replicates). **c** Immunoprecipitation/immunoblot analysis of the WCLs indicated anti-Myc derived from control, ALKBH1 WT or 233A overexpression 293 T cells transfected with Myc-OST48, IgG served as the negative controls (*n* = 3 biological replicates). **d** Immunoprecipitation/immunoblot analysis of the WCLs indicated anti-Myc derived from control and ALKBH1 knockdown 293 T cells transfected with Myc-OST48, IgG served as the negative controls (*n* = 3 biological replicates). **e, f** Western blots (**e**) and quantitative results (**f**) indicated proteins in control and ALKBH1 knockdown HeLa cells after treatment with 10 μM NGI-1 for 24 h (*n* = 4 independent samples). **g, h** Representative images (**g**) and quantitative results (**h**) of PAmCherry puncta (red) in control and ALKBH1 knockdown HeLa cells stably expressing PAmCherry-KFERQ-NE under serum deprivation for 16 h after 10 μM NGI-1 pretreatment for 24 h (Scale bar = 10 μm, *n* = 20 independent cells). **i-k** Representative images (**i**) and quantitative results indicated the number of Gal3-EGFP puncta (**j**) and relative Feret diameter (**k**) in control and ALKBH1 knockdown HeLa cells stably expressing Gal3-EGFP under 10 μM NGI-1 pretreatment for 24 h and 1 mM LLOMe treatment for 60 min (Scale bar = 10 μm, n = 25 independent cells; Scale bar = 2 μm by zoomed in, *n* = 35 independent puncta). Data are presented as mean values ± SD; *p < 0.05, **p < 0.001, ***p < 0.001; ns indicated no significance

Next, we investigated how ALKBH1 regulates the glycosylation of LAMPs. As the impact of ALKBH1 on the glycosylation of LAMPs was independent of its demethylation enzymatic activity, we hypothesized that ALKBH1 affects the degree of glycosylation through protein interactions. To address this, a co*-*immunoprecipitation/mass spectrometry (Co*-*IP/MS) assay was employed to identify proteins that potentially interact with ALKBH1. Among the interacting proteins, we observed OST48 (Supplementary Fig. 6a), a constituent of the OST complex, which enables the transfer of preassembled oligosaccharides to specific asparagine residues for the generation of *N-*glycosylation [33]. Subsequently, the interaction between ALKBH1 and OST48 was verified through co*-*immunoprecipitation assays (Fig. 6a, b). We further evaluated the impact of ALKBH1 on the protein expression of the OST complex. Immunoblotting analysis revealed that the protein levels of three pivotal components of the OST complex remained unchanged with ALKBH1 knockdown (Supplementary Fig. 6b).

Previous studies have pointed out that the structural integrity of OST complexes is crucial for their catalytic function [63]. Thus, we hypothesized that ALKBH1 competitively interacts with OST48, leading to structural disruption of the OST complex. Indeed, we found that the interaction between OST48 and STT3A or DAD1 was attenuated following ALKBH1 overexpression (Fig. 6c). Conversely, silencing of ALKBH1 led to a strengthened interaction between OST48 and STT3A or DAD1, along with reduced interactions with ALKBH1 and OST48 (Fig. 6d). These results consistently indicate that ALKBH1 competitively binds to OST48, which impaired the structural integrity among subunits of OST complex.

Recently, a small-molecule *N-*linked glycosylation inhibitor 1 (NGI-1) has been identified to partially inhibit *N-*glycosylation by targeting OST complexes [64]. Treatment of HeLa cells with 10 μM NGI-1 for 24 h resulted in the severe blockage of LAMP1 and LAMP2A glycosylation, which was similar to the effects observed with ALKBH1 overexpression (Fig. 6e). Meanwhile, the protein levels of HIF-1*α* were rescued in ALKBH1 knockdown cells following NGI-1 treatment (Fig. 6e, f). Furthermore, we observed that the increased CMA activity resulting from ALKBH1 depletion, as measured by the CMA reporter, was attenuated after NGI-1 treatment (Fig. 6g, h). Subsequently, the lysosomes labeled by LAMP1 exhibited an increase in mean fluorescence intensity (MFI) and volume upon NGI-1 treatment, which was comparable to the lysosomal swelling caused by ALKBH1 overexpression (Supplementary Fig. 6c–e). In addition, the swelling of lysosomal compartments was also observed using the Gal3-EGFP reporter under LLOMe treatment, and the growing number of Galectin 3 represented the deterioration of LMP (Fig. 6i–k).

To further verify whether the destruction of the OST48 complex affects the degradation of HIF-1*α* through CMA, stable knockdown of OST48 cells was generated by lentivirus-containing shRNA. Immunoblotting revealed that OST48 depletion significantly impaired the glycosylation of LAMP1 and LAMP2A, concomitant with a marked rescue of HIF-1*α* levels in ALKBH1 knockdown cells (Supplementary Fig. 7a, b). In addition, the depletion of OST48 attenuated the heightened CMA activity resulting from ALKBH1 depletion, as determined by the CMA reporter (Supplementary Fig. 7c, d). Moreover, it was noted that OST48 silencing led to an increased presence of Galectin 3 and the occurrence of swollen lysosome morphology, akin to the phenomenon observed upon NGI-1 treatment (Supplementary Fig. 7e–g). Collectively, this evidence indicated that ALKBH1 affects *N-*glycosylation by interfering with the structural integrity of OST complex.

## Discussion

**Fig. 7:**
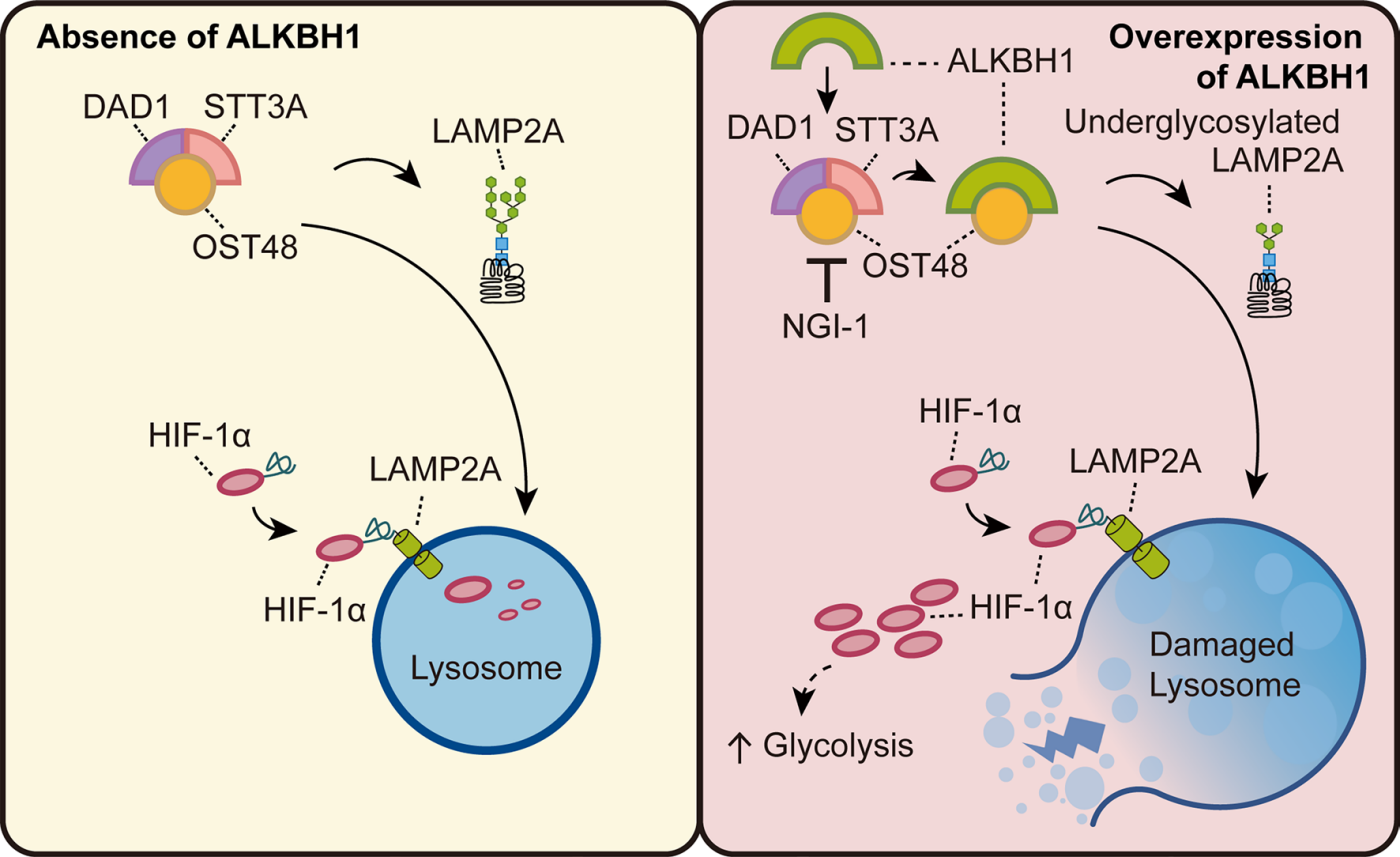
Model of ALKBH1 promotes HIF-1*α*-mediated glycolysis by inhibiting *N-*glycosylation of LAMP2A. The absence of ALKBH1 enhances the glycosylation of LAMP2A, thereby accelerating the degradation of HIF-1*α* via CMA (left panel). Excessive ALKBH1 or NGI-1 disrupting the structural integrity among subunits (STT3A and DAD1) of the OST complex, resulting in underglycosylated LAMP2A, as well as damaged lysosomal homeostasis and impeded CMA-mediated degradation of HIF-1*α*. Accumulated HIF-1*α* then promotes the expression of glycolysis-related genes (right panel)

In this study, we found that the absence of ALKBH1 enhanced the glycosylation of LAMP2A, thereby accelerating the degradation of HIF-1*α* via CMA. Conversely, an excess of ALKBH1 competitively binds to OST48, disrupting the structural integrity among subunits of the OST complex, resulting in the generation of underglycosylated LAMP2A. Hereafter, LAMP2A with defective *N-*glycosylation triggered lysosomal dysfunction, including lysosomal swelling, high lysosomal pH, and vulnerable membrane permeability. In addition, abnormal glycosylation of LAMP2A prevented the degradation of HIF-1*α* via CMA. Eventually, the accumulated HIF-1*α* transferred to the nucleus to promote the expression of glycolysis-related genes (Fig. 7).

Previous studies described that ALKBH1 can respond to glucose availability, a phenomenon mainly manifested by the upregulation of ALKBH1 expression under conditions of glucose deprivation [13]. Nevertheless, the question of whether ALKBH1 actively modulates glucose metabolism and the underlying molecular mechanisms remains to be fully understood. In our present study, we observed that depletion of ALKBH1 resulted in reduced production of pyruvate and lactate, as well as decreased glycolysis enzymes and extracellular acidification rate. These alterations are predominantly caused by HIF-1*α*, as the reintroduction of this factor significantly reverses the decreased glycolytic activity (Fig. 1). Numerous investigations have underscored the pivotal role of ALKBH1 in carcinogenesis. ALKBH1-mediated 6 mA demethylation has been found to selectively target NRF1 transcriptional activity, subsequently inactivating AMPK signaling and promoting the “Warburg” phenotype [11]. Otherwise, it has been reported that depletion of ALKBH1 in MSCs and 3T3-L1 cells resulted in decreased mRNA levels of HIF-1*α*, as ALKBH1 is involved in the DNA demethylation at the 6 mA site of HIF-1*α* during adipose differentiation [9]. HIF-1*α* mediating the reprogramming of the glucose metabolic pathway in cancer cells has become a characteristic of general attention, especially on the Warburg effect, making it a potential therapy to face the progression of cancers. Contrary to these findings, our study did not observe significant differences in the transcription level of HIF-1*α* following either ALKBH1 knockdown or overexpression cells (Fig. 1e; Supplementary Fig. 1b). It is worth noting that the reported differences in localizations of ALKBH1 between terminally differentiated and undifferentiated cells may contribute to this discrepancy [5, 65], and further investigations should be conducted in future studies to explore the variations among different cell lines.

HIF-1*α* is a prototypical oxygen*-*sensitive dimeric transcription factor that undergoes rapid turnover within cells under normoxic conditions [66, 67]. Numerous studies have shown that HIF-1*α* is mainly regulated through its degradation pathway. In our investigation, the knockdown of ALKBH1 led to a decrease in the protein level of HIF-1*α*, mainly through lysosome-dependent degradation rather than through a proteasome-dependent mechanism. Although it has been established that HIF-1*α* degradation is primarily mediated by ubiquitination, our experimental observations revealed that the application of various ubiquitination inhibitors did not succeed in inhibiting HIF-1*α* degradation after ALKBH1 knockdown. In contrast, the use of inhibitors targeting lysosomal degradation efficiently prevented the degradation of HIF-1*α* (Fig. 2; Supplementary Fig. 2). CMA is a lysosome-dependent protein degradation pathway that accelerates activation in the face of cellular stress such as serum starvation [68]. Recent evidence indicates that the degradation of HIF-1*α* may occur by CMA in an oxygen*-*independent manner [50, 69]. After excluding the effects of macroautophagy, we demonstrated that ALKBH1 promotes glycolysis via suppressing CMA-mediated degradation of HIF-1*α* (Fig. 3). ALKBH1 has been extensively studied and demonstrated to conduct demethylation in various nucleic acids [70]. The active site pockets of ALKBH1 are critical for its catalytic activity. The D233A mutation in ALKBH1 has been established to significantly impair its demethylase activity on both DNA and RNA substrates [2, 3]. However, our study reveals that the involvement of ALKBH1 in CMA is not dependent on its enzymatic activity.

Mounting evidence demonstrates a functional relationship between macroautophagy and CMA, dysfunction of either one of them can lead to a compensatory upregulation of the other, which helps cells to resist various types of damage to maintain cell viability [71, 72]. In the present study, we observed that silencing ALKBH1 promotes CMA while hindering the initiation of macroautophagy, potentially resulting from macroautophagy–CMA crosstalk. However, further substantiating evidence is required to support this hypothesis. Otherwise, although evidence exists for the regulatory roles of AlkB family members on autophagy, the impact of ALKBH1 on this process remains unexplored. For instance, it has been reported that ALKBH5 can be regulated by TFEB, leading to the activation of the AMPK-ULK1 axis and autophagy occurrence [73]. In addition, FTO has been shown to upregulate Atg5 and Atg7 expression via a YTHDF2-mediated pathway, thereby promoting autophagy in mouse pre-adipose cell lines [74]. In the present study, we observed that the silencing ALKBH1 impedes the initial phases of autophagy, particularly resulting in a substantial reduction in the expression of genes involved in the autophagic elongation phase (Supplementary Fig. 8). Nonetheless, considering the variations in the significance of regulatory mechanisms across different models, the role of ALKBH1 in autophagy still needs further investigation.

Due to the low pH characteristics of lysosomes, a variety of glycosylated proteins are involved in lysosomal membrane stability and fusion of lysosomes with other organelles [75]. These proteins are glycosylated on both asparagine residues (*N-*linked) and serine or threonine residues (*O-*linked) within their luminal domains [76]. *N-*linked glycosylation initially occurs in the ER lumen through a membrane-associated enzyme complex known as the OST complex [77]. Previous research has emphasized the vital role of the structural integrity of OST complexes in their catalytic function, with the depletion of any subunits leading to severe hypo*-*glycosylation phenomenon [63]. Our results indicate that ALKBH1 knockdown increases *N-*linked glycosylation of LAMPs, as confirmed by glycosylase cleavage enzymes and *O-*linked glycosylation antibodies assays (Fig. 4). Moreover, we identify that ALKBH1 competitively interacts with OST48, resulting in the disturbance of the OST48 complex’s structural integrity and ultimately causing glycosylation defects in LAMPs (Fig. 6). Notably, the depletion of OST48 significantly impaired the glycosylation of LAMP1 and LAMP2A, concomitant with a marked rescue of HIF-1*α* levels in ALKBH1 knockdown cells (Supplementary Fig. 7a, b). This conclusion aligns with a recent report, which has highlighted that the depletion of OST48 leads to the inhibition of cell surface *N-*glycosylation, potentially associated with the development of congenital disorders of glycosylation (CDG) [36]. Moreover, it has been shown that abnormal LAMP1 glycosylation plays a role in NPC pathology [37], which is a fatal neurodegenerative disorder caused by an accumulation of free cholesterol and glycosphingolipids in the lysosome [78]. This suggests that ALKBH1 might play a role in NPC pathology and could be a potential therapeutic target.

Several research reports suggest that deglycosylated LAMP1 and LAMP2 undergo rapid degradation by proteases in their respective environment, leading to various morphological alterations, notably the presence of acidic swollen vacuoles [31]. In light of the alterations in LAMPs’ glycosylation induced by ALKBH1, we further explored the homeostasis and functionality of lysosomes and revealed that overexpression of ALKBH1 increased the lysosomal abundance within cells, concomitantly in compromised lysosomal morphology and functionality (Fig. 5). Furthermore, we have observed that ALKBH1 has a substantial impact on the maturation of lysosomal hydrolases and the subcellular localization of TFEB, a pivotal transcription factor governing lysosomal biogenesis (Supplementary Fig. 9). These findings align with prior evidence illustrating an elevation in lysosomal pH and an increased lysosomal abundance within cells following ALKBH1 overexpression. A multitude of kinases or phosphatases have been reported to regulate the phosphorylation status of TFEB via diverse mechanisms, including ERK2, MTORC1, GSK3*β*, Akt, PKC, PP2A, calcineurin, and GCN5 [79]. Given the current uncertainty regarding the direct regulatory role of a specific kinase in the cytoplasmic–nucleus shuttling of TFEB induced by ALKBH1, or whether it is an indirect consequence of lysosomal damage, additional research is warranted to elucidate the influence of ALKBH1 on the cellular distribution of TFEB.

NGI-1, a small-molecule inhibitor specifically targeting the OST complex, was initially discovered for its ability to inhibit the flaviviruses [80]. Recently, NGI-1 has been reported to affect the *N-*glycopeptide of LAMP2, triggering lysosomal defects and autophagy [81]. In this study, we observed that NGI-1 can inhibit the glycosylation of LAMPs and rescue the degradation of HIF-1*α* caused by ALKBH1 knockdown (Fig. 6e-f). In addition, treatment with NGI-1 impaired CMA activity and lysosomal membrane permeability, similar to the effects observed during ALKBH1 overexpression (Fig. 6g-k). Furthermore, it has been reported that NGI-1 can effectively block EGFR signal transduction by inhibiting *N-*glycosylation of EGFR in tyrosine kinase inhibitor (TKI) resistance non*-*small cell lung cancer (NSCLC), leading to cell-cycle arrest and proliferative block [82]. These findings highlight the potential of targeting *N-*glycosylation of glycoproteins as a novel approach in cancer therapy.

In conclusion, our study proposes a regulatory mechanism by which ALKBH1 regulates glycolysis via HIF-1*α* signaling in a demethylase-independent manner. Mechanistically, excessive ALKBH1 competitively binds to the OST48, leading to impaired structural integrity of the OST complex and subsequent defective *N-*glycosylation of LAMPs, particularly LAMP2A. Abnormal glycosylation of LAMP2A disrupts lysosomal homeostasis and impedes CMA-mediated degradation of HIF-1*α*. Eventually, accumulated HIF-1*α* translocates into the nucleus and activates downstream glycolysis-related genes.

### Supplementary Information

Below is the link to the electronic supplementary material.

Supplementary file1 (PDF 3577 kb)

## Data Availability

All data supporting this study are available from the corresponding authors upon reasonable request.

## Abbreviations

ALKBH1: AlkB homolog 1
OST: Oligosaccharyltransferase
CMA: Chaperone-mediated autophagy
HIF-1*α*: Hypoxia-inducible factor 1 subunit alpha
HK2: Hexokinase 2
PKM2: Pyruvate kinase M2
PGK1: Phosphoglycerate kinase 1
LDHA: Lactate dehydrogenase A
PHDs: Prolyl hydroxylase domain proteins
VHL: Von Hippel–Lindau
HREs: Hypoxia-response elements
HSC70: Heat shock cognate protein 70
LAMP2A: Lysosome-associated membrane protein type 2A
NPC: Niemann–Pick disease, type C
ECAR: Extracellular acidification rate
6-AN: 6-Aminonicotinamide
Endo H: Endoglycosidase H
PNGase F: Peptide-*N-*glycosidase F
*O-*GlcNAc: *O-*Linked *N-*acetylglucosamine
MFI: Mean fluorescence intensity
LMP: Lysosomal membrane permeabilization
Gal3-EGFP: EGFP-tagged Galectin 3
NGI-1: *N-*Linked glycosylation inhibitor 1
CDG: Congenital disorders of glycosylation
