# Supplementary material for: ALKBH1 promotes HIF-1α-mediated glycolysis by inhibiting N-glycosylation of LAMP2A
Source: Cell Mol Life Sci. 2024 Mar 12;81(1):130. doi: 10.1007/s00018-024-05152-z (PMC10933178; doi:10.1007/s00018-024-05152-z)
Supplement: Supplementary file 1 — Supplementary file1 (PDF 3577 kb) [file 18_2024_5152_MOESM1_ESM.pdf]

Supplementary for ALKBH1 promotes HIF-1α-mediated glycolysis by inhibiting *N*-glycosylation of LAMP2A

Supplementary Figures

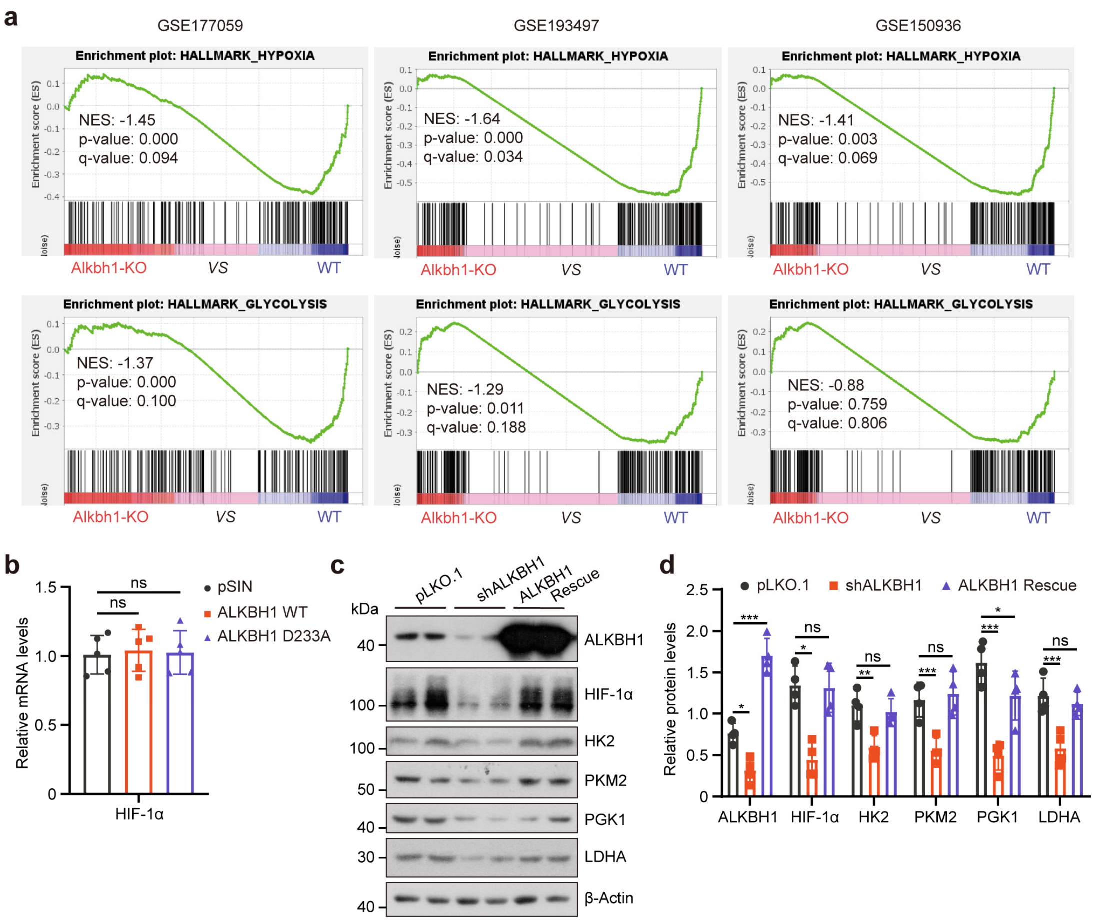

Supplementary Figure 1 ALKBH1 involves in the regulation of glycolysis via HIF-1α.

a Gene set enrichment analysis (GSEA) shows hypoxia and glycolysis signaling pathway enriched in ALKBH1 knockout groups. **b** Relative transcript levels of HIF-1α were analyzed by q-PCR in control, ALKBH1 WT and ALKBH1 D233A overexpression HeLa cells; ACTB was used as a control (n = 5 independent samples). **c, d** Western blots (**c**) and quantitative results (**d**) of the indicated proteins in control, ALKBH1 knockdown and ALKBH1 knockdown HeLa cells transfected with exogenous ALKBH1 (n = 4 independent samples). Data were presented as mean values ± SD; *$p < 0.05$, **$p < 0.001$, ***$p < 0.001$; ns indicated no significance.

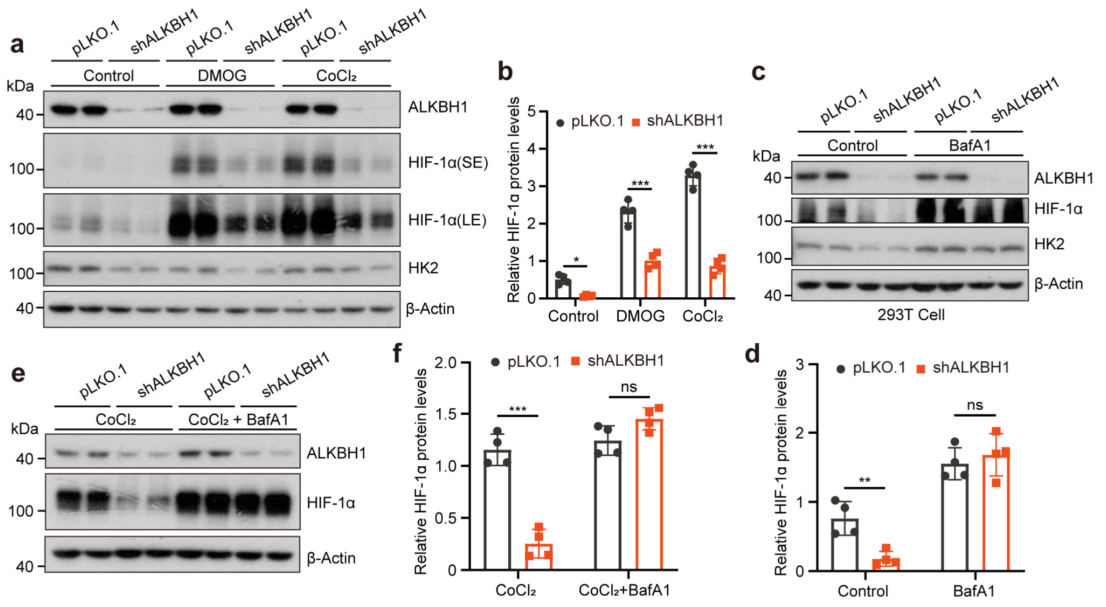

Supplementary Figure 2 Depletion of ALKBH1 promotes the lysosomal degradation of HIF-1α.

a, b Western blots (**a**) and quantitative results (**b**) of the indicated proteins in control and ALKBH1 knockdown HeLa cells treated with 100 μM dimethyloxalylglycine (DMOG) or 200 nM cobalt chloride ($CoCl_2$) for 12 h (n = 4 independent samples), (SE) indicates short-term exposure and (LE) indicates short-term exposure. **c, d** Western blots (**c**) and quantitative results (**d**) of the indicated proteins in control and ALKBH1 knockdown 293T cells treated with 100 nM Bafilomycin A1 (BafA1) for 12 h (n = 4 independent samples). **e, f** Western blots (**e**) and quantitative results (**f**) of the indicated proteins in control and ALKBH1 knockdown HeLa cells treated with 200 nM $CoCl_2$ or a combination of $CoCl_2$ and BafA1 for 12 h (n = 4 independent samples). Data were presented as mean values ± SD; *$p < 0.05$, **$p < 0.001$, ***$p < 0.001$; ns indicated no significance.

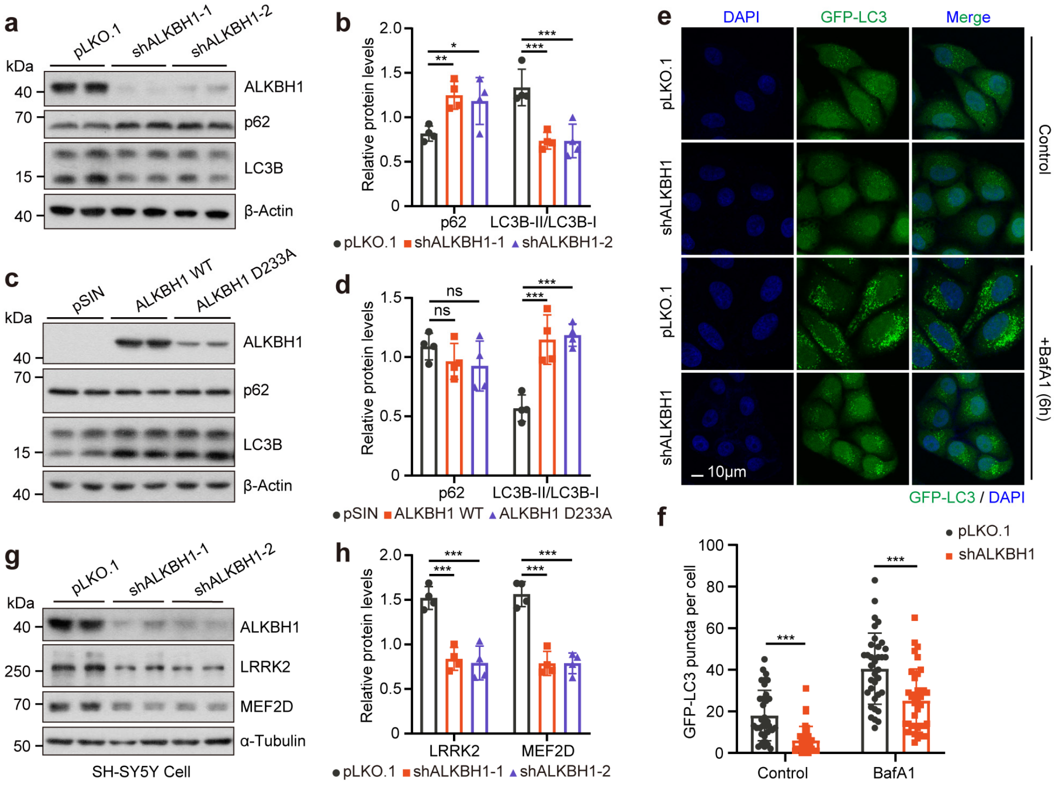

Supplementary Figure 3 ALKBH1 silencing mediates degradation of HIF-1α independent macroautophagy.

a, b Western blots (**a**) and quantitative results (**b**) reflected the expression of autophagy-related proteins in control and ALKBH1 knockdown HeLa cells (n = 4 biological replicates). **c, d** Western blots (**c**) and corresponding quantitative results (**d**) revealed autophagy-related proteins in control, ALKBH1 WT or ALKBH1 D233A overexpression HeLa cells (n = 4 biological replicates). **e, f** Representative images (**e**) and quantitative results (**f**) reflected GFP-LC3 puncta (green) per cell in control and ALKBH1 knockdown cells stably expressing GFP-LC3 with or without 100 nM BafA1 for 6 h (Scale bar = 10 μm; n = 35 independent cells). **g, h** Western blots (**g**) and quantitative results (**h**) of the indicated proteins in control and ALKBH1 knockdown SH-SY5Y cells (n = 4 biological replicates). Data were presented as mean values ± SD; *p < 0.05, **p < 0.001, ***p < 0.001; ns indicated no significance.

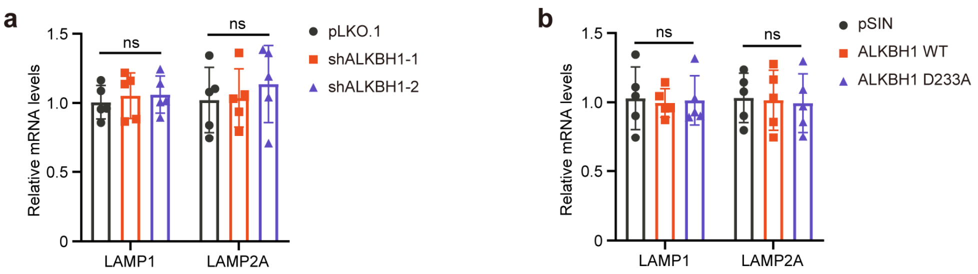

Supplementary Figure 4 ALKBH1 does not affect the transcriptional expression of LAMPs.

a Relative transcript levels of the indicated gene were analyzed by q-PCR in control and ALKBH1 knockdown HeLa cells; ACTB was used as a control (n = 5 independent samples). **b** Relative transcript levels of the indicated gene were analyzed by q-PCR in control, ALKBH1 WT or 233A overexpression HeLa cells; ACTB was used as a control (n = 5 independent samples). Data were presented as mean values ± SD; **p* < 0.05, ***p* < 0.001, ****p* < 0.001; ns indicated no significance.

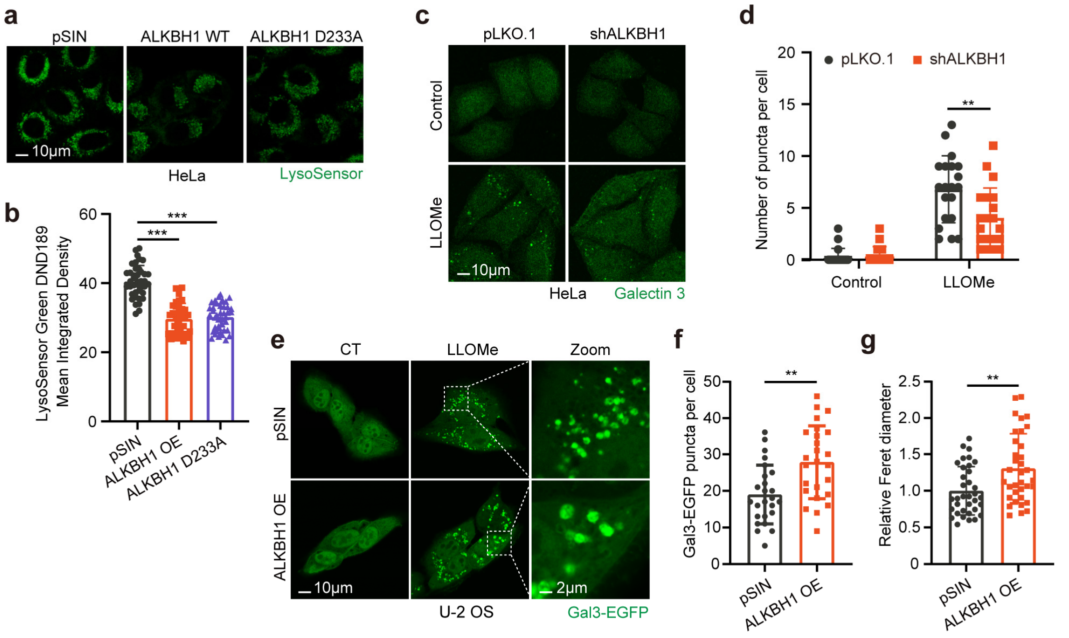

Supplementary Figure 5 Overexpression of ALKBH1 triggers dysfunction of lysosome.

a, b Representative images (**a**) and quantitative results (**b**) revealed the mean integrated density of LysoSensor Green in control, ALKBH1 WT or D233A overexpression HeLa cells (Scale bar = 10 μm; n = 40 independent cells). **c, d** Representative images (**c**) and quantitative results indicated the number of endogenous Galectin 3 puncta (**d**) in control and ALKBH1 knockdown cells with or without 1 mM LLOMe treatment for 60 min (Scale bar = 10 μm; n = 20 independent cells). **e-g** Representative images (**e**) and quantitative results indicated the number of Gal3-EGFP puncta (**f**) and relative Feret diameter (**g**) in control and ALKBH1 overexpression U-2 OS cells stably expressing Gal3-EGFP with or without 1 mM LLOMe treatment for 60 min (Scale bar = 10 μm, n = 25 independent cells; Scale bar = 2 μm by zoomed in, n = 35 independent puncta). Data were presented as mean values ± SD; *$p < 0.05$, **$p < 0.001$, ***$p < 0.001$; ns indicated no significance.

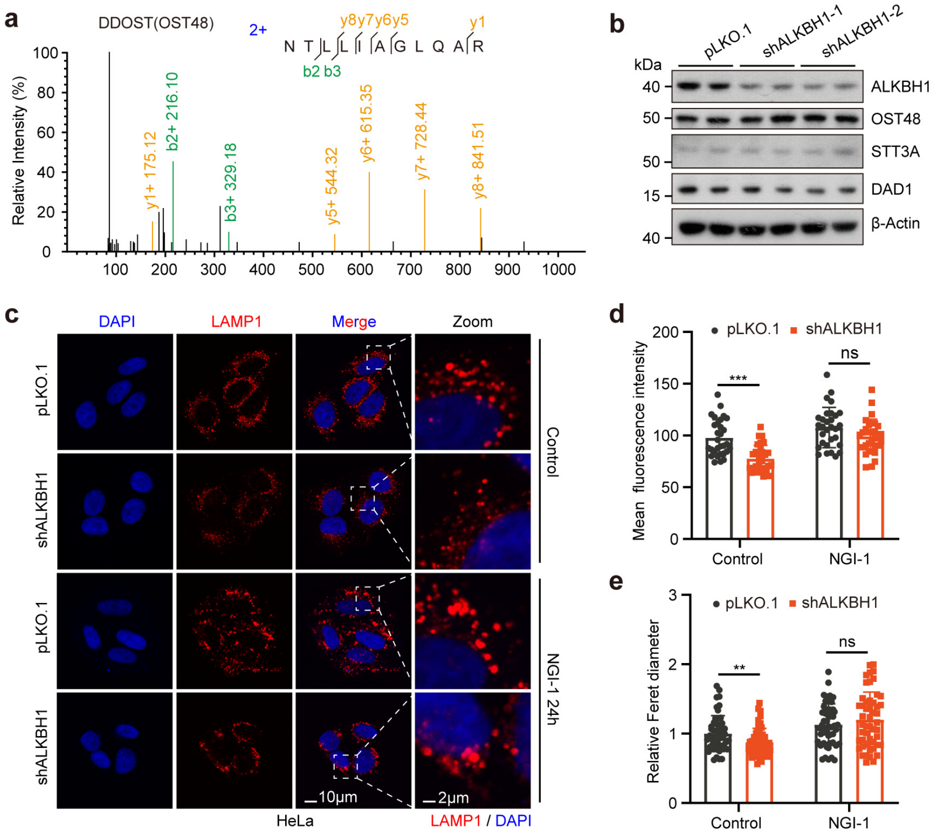

Supplementary Figure 6 ALKBH1 affects *N*-glycosylation by interfering with the OST complex.

a Mass spectrometry (MS) analysis of DDOST/OST48 peptide. **b** Western blots reflected the pivotal components of the OST complex in control and ALKBH1 knockdown HeLa cells. **c-e** Representative images (**c**) and quantitative results revealed the mean fluorescence intensity (**d**) and relative Feret diameter (**e**) labeled by anti-LAMP1 (red) in control and ALKBH1 knockdown HeLa cells after treatment with 10 μM NGI-1 for 24h (Scale bar = 10 μm, n = 30 independent cells; Scale bar = 2 μm by zoomed in, n = 50 independent puncta). Data were presented as mean values ± SD; **p* < 0.05, ***p* < 0.001, ****p* < 0.001; ns indicated no significance.

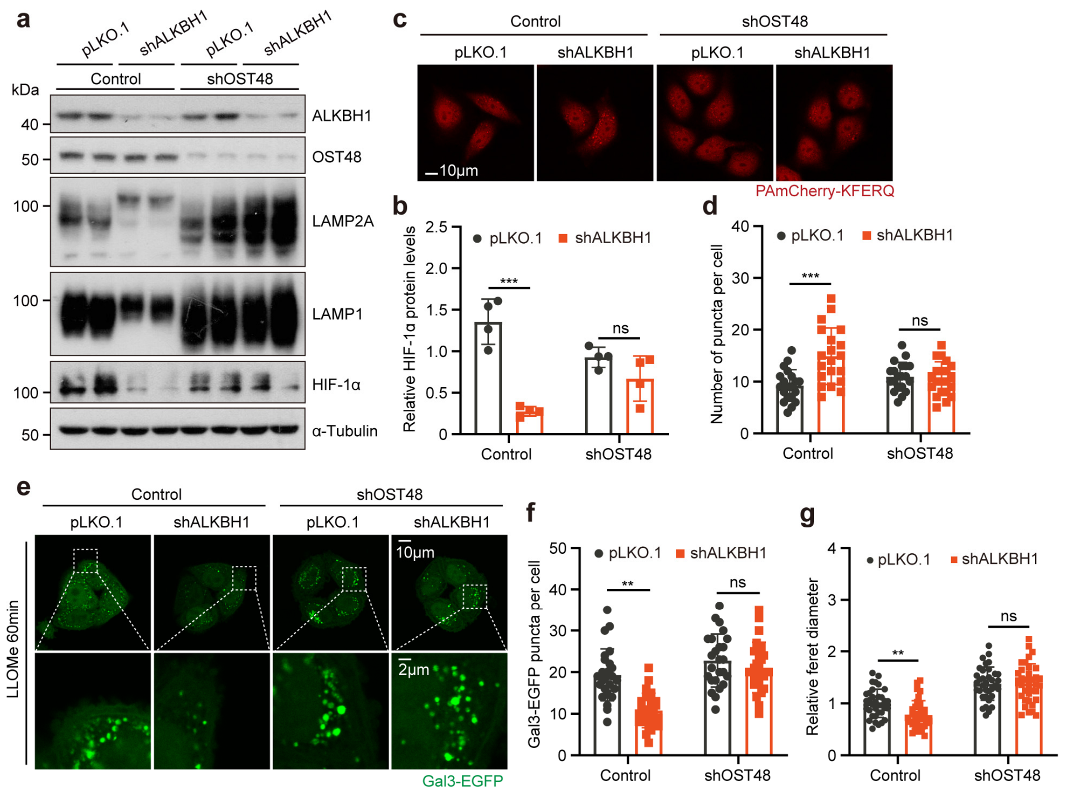

Supplementary Figure 7 OST48 depletion suppresses *N*-glycosylation of LAMPs and disrupts lysosomal homeostasis.

a, b Western blots (**a**) and quantitative results (**b**) indicated the HIF-1α protein expression in control, ALKBH1 knockdown or ALKBH1, OST48 double knockdown HeLa cells (n = 4 biological replicates). **c, d** Representative images (**c**) and quantitative results (**d**) of PAmCherry puncta (red) in control, ALKBH1 knockdown or ALKBH1, OST48 double knockdown HeLa cells stably expressing PAmCherry-KFERQ-NE under serum deprivation for 16 h (Scale bar = 10 μm; n = 20 independent cells). **e-g** Representative images (**e**) and quantitative results indicated the number of Gal3-EGFP puncta (**f**) and relative Feret diameter (**g**) in control, ALKBH1 knockdown or ALKBH1, OST48 double knockdown HeLa cells stably expressing Gal3-EGFP under 1mM LLOMe treatment for 60 min (Scale bar = 10 μm, n = 25 independent cells; Scale bar = 2 μm by zoomed in, n = 35 independent puncta). Data were presented as mean values ± SD; *$p < 0.05$, **$p < 0.001$, ***$p < 0.001$; ns indicated no significance.

a

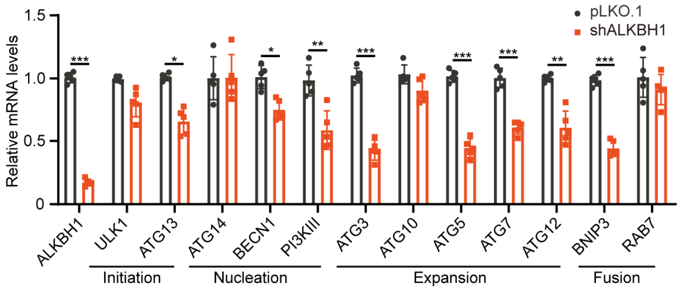

Supplementary Figure 8 Knockdown of ALKBH1 inhibits the expression of autophagy-related genes.

a Relative transcript levels of the autophagy-related gene were analyzed by q-PCR in control and ALKBH1 knockdown HeLa cells; ACTB was used as a control (n = 5 independent samples). Data were presented as mean values ± SD; **p* < 0.05, ***p* < 0.001, ****p* < 0.001; ns indicated no significance.

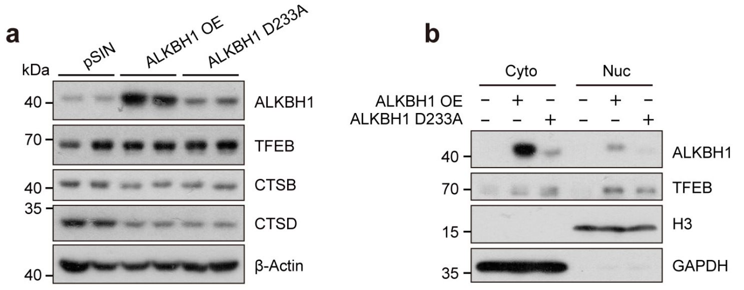

Supplementary Figure 9 Overexpression of ALKBH1 affects the expression of lysosomal hydrolases and the nuclear translocation of TFEB.

a Western blot reflected the indicated proteins in control, ALKBH1 WT and D233A overexpression HeLa cells. **b** Nuclear and cytoplasmic separation assay and western blotting analysis for TFEB in control, ALKBH1 WT and D233A overexpression HeLa cells.

Supplementary Table

Supplementary Table 1 Primary and secondary antibodies used in the present study.

**Antibody Name (Primary antibodies)**	**Company**	**Item number**	**Application**
ALKBH1 Polyclonal antibody	Proteintech	27973-1-AP	WB, 1:2000
ALKBH1 Rabbit pAb	ABclonal	A14079	IP, 1:500
HIF-1α Rabbit mAb	CST	#36169S	WB, 1:1000
Hexokinase 2 Polyclonal antibody	Proteintech	22029-1-AP	WB, 1:3000
PKM2-specific Polyclonal antibody	Proteintech	15822-1-AP	WB, 1:3000
PGK1 Polyclonal antibody	Proteintech	17811-1-AP	WB, 1:3000
LDHA-Specific Polyclonal antibody	Proteintech	19987-1-AP	WB, 1:3000
β-Actin Mouse monoclonal	Sigma	A1978	WB, 1:4000
α-Tubulin Rabbit mAb	CST	#2125	WB, 1:3000
LC3B antibody	Sigma	L7543	WB, 1:2000
p62/SQSTM1 antibody	Sigma	P0067	WB, 1:3000
Monoclonal ANTI-FLAG® M2	Sigma	F1804	IP, 1:1000
LAMP2A	Abcam	ab125068	WB, 1:1000
LAMP1 Rabbit mAb	CST	#9091	WB, 1:3000<br>IF, 1:200
Hsc70 Polyclonal antibody	Protientech	10654-1-AP	WB, 1:3000
Anti-O-Linked N-Acetyl-glucosamine Rabbit mAb	PTM BIO	PTM-951RM	IP, 1:1000
Galectin-3 Monoclonal antibody	Protientech	60207-1-Ig	IF, 1:200
MYC tag Polyclonal antibody	Protientech	16286-1-AP	IP, 1:1000

DDOST/OST48 Polyclonal antibody	Protientech	14916-1-AP	IP, 1:2000
STT3A Polyclonal antibody	Protientech	12034-1-AP	IP, 1:2000
DAD1 Polyclonal antibody	Protientech	10531-1-AP	IP, 1:2000
LRRK2 Rabbit pAb	ABclonal	A17253	WB, 1:2000
MEF2D Polyclonal antibody	Protientech	14353-1-AP	WB, 1:2000
Cathepsin B Polyclonal antibody	Protientech	12216-1-AP	WB, 1:2000
Cathepsin D Polyclonal antibody	Protientech	55021-1-AP	WB, 1:2000
TFEB Rabbit pAb	ABclonal	A7311	WB, 1:2000
**Antibody Name (Secondary antibodies)**	**Company**	**Item number**	**Application**
Peroxidase AffiniPure Goat Anti-Rabbit IgG	Jackson ImmunoResearch	111-035-144	WB, 1:5000-1:10000
Peroxidase AffiniPure Goat Anti-Mouse IgG	Jackson ImmunoResearch	115-035-146	WB, 1:5000-1:10000
Alexa Fluor®594-AffiniPure goat anti-rabbit IgG	Jackson ImmunoResearch	115-585-146	IF, 1:200-400
Alexa Fluor®488-AffiniPure goat anti-mouse IgG	Jackson ImmunoResearch	115-545-146	IF, 1:200-400
Mouse Anti-Rabbit IgG (Light-Chain Specific) (D4W3E)	CST	#93702	IP, 1:2000
VeriBlot for IP Detection Reagent (HRP)	Abcam	ab131366	IP, 1:2000

Supplementary Table 2 qPCR primers sequences used in the present study.

**Gene**	**Forward Primer, 5' to 3'**	**Reverse Primer, 5' to 3'**
ALKBH1	ATGGACTCAAAGGCTATCCTGG	GTCTCCGTTTAGTCGCTTCTTTA
HIF-1α	ATCCATGTGACCATGAGGAAATG	TCGGCTAGTTAGGGTACACTTC
HK2	TGCCACCAGACTAAACTAGACG	CCCGTGCCCACAATGAGAC
PKM2	ATGTCGAAGCCCCATAGTGAA	TGGGTGGTGAATCAATGTCCA
PGK1	GAACAAGGTTAAAGCCGAGCC	GTGGCAGATTGACTCCTACCA
LDHA	TTGACCTACGTGGCTTGGAAG	GGTAACGGAATCGGGCTGAAT
LAMP1	CAGATGTGTTAGTGGCACCCA	TTGGAAAGGTACGCCTGGATG
LAMP2A	AGACTGTTTCAGTGTCTGGAGC	CGCTATGGGCACAAGGAAGTT
ULK1	AAGTTCGAGTTCTCTCGCAAG	CGATGTTTTCGTGCTTTAGTTCC
ATG13	CCAGGCTCGACTTGGAGAAAA	AGATTTCCACACACATAGATCGC
ATG14	GAGGGCCTTTACGTGGCTG	AATAGACGAAATCACCGCTCTG
BECN1	ATGGAGGGGTCTAAGGCGTC	TCCTCTCCTGAGTTAGCCTCT
PI3KIII	AAAGGCCGAGCCCTCTATTAT	GGACAATCTCGACGTAAGAAGC
ATG3	TTCAGTTCACCCATGCAGGC	GTTAACAGCCATTTTGCCACTAA
ATG10	GTAGTTACCAAGTGCCGGTTC	AGCTAACGGTCTCCCATCTAAA
ATG5	AGCCAGGTGATGATTCACGG	GGCTGGGGGACAATGCTAA
ATG7	GTTCGCCCCCTTTAATAGTGC	TGAACTCCAACGTCAAGCGG
ATG12	CCAAGGACTCATTGACTTCATC	CAGTAATGCAGGACCAGTTTACC
BNIP3	TCCTGGGTAGAACTGCACTTC	GCTGGGCATCCAACAGTATTT
RAB7	GGTTCCGCTCAATGGTATCAA	TGCCCTGGTACTTCCTATCTTC
ACTB	CATGTACGTTGCTATCCAGGC	CTCCTTAATGTCACGCACGAT
